# Loose parts play encourages spontaneous science, technology, engineering, and mathematics (STEM) behaviours

**DOI:** 10.1038/s44271-025-00362-y

**Published:** 2025-12-05

**Authors:** Ozlem Cankaya, Natalia Rohatyn-Martin, Karen Buro, Okan Bulut, Keirsten Taylor

**Affiliations:** 1 Department of Early Learning and Curriculum Studies, MacEwan University, Edmonton, AB Canada; 2 Department of Health and Human Services, MacEwan University, Edmonton, AB Canada; 3 Department of Mathematics and Statistics, MacEwan University, Edmonton, AB Canada; 4 Department of Educational Psychology, University of Alberta, Edmonton, AB Canada; 5 Department of Educational Psychology, University of Calgary, Calgary, AB Canada

**Keywords:** Human behaviour, Education

## Abstract

Children incorporate items found in their environment into their play, transforming everyday objects and materials into an opportunity for exploration. Termed loose parts, these versatile, natural, or manufactured materials (e.g., cardboard, pipes, buttons, sticks) are widely recommended for supporting children’s early STEM learning. Limited empirical work has documented children’s indoor STEM behaviours with loose parts. Using a within-subjects experimental design, we examined children’s early STEM behaviours and engagement (*N* = 60; 32 females, 28 males; *Mage* = 58.6 months, *SD* = 10.9) during unstructured solitary play with loose parts and toys that have limited function and affordance (e.g., toy percussion instruments; control). We conducted observations of children’s STEM behaviours. Children’s cognitive functioning, executive function, and home learning environment were also assessed via standardized measures and parent reports. Children demonstrated significantly more STEM behaviours with loose parts than in the control condition. There was no credible evidence that these behaviours differed by sex. Cognitive functioning predicted STEM Engagement Score with loose parts, with children’s verbal comprehension being the strongest predictor in the control condition. Children’s executive function and parents’ attitudes regarding play and engagement in play activities at home predicted constructing structures, which were the most common STEM behaviours. This study thus demonstrates that loose parts may offer a powerful opportunity for STEM-related early learning; however, children’s cognitive capacities and home experiences should be considered, rather than assuming uniform benefits.

## Abstract (web summary)

Using a within-subjects experimental design, children’s early STEM behaviours during solitary play with loose parts (e.g., acorn, cardboard) were examined with findings indicating that play with loose parts offers strong potential for STEM exploration and learning.

## Introduction

Play is a natural, effective entry point for young children’s foundational science, technology, engineering, and mathematics (STEM) engagement and learning that can foster innovative thinking^1–6^. In play, children explore, test hypotheses, and solve problems, building knowledge through interactions with their environment^7–11^. In particular, unstructured play allows children to observe, manipulate materials, plan, test ideas, and solve problems independently^12–14^.

Educators and researchers recognize the value of integrating curricular goals with play-based learning^3,15,16^. A promising approach is using everyday materials and objects, known as *loose parts*, to enrich children’s play. Loose parts are natural or manufactured objects or materials that are not toys but that children can repurpose during play. Loose parts are endorsed for their potential to support STEM learning and innovation^17–20^. However, children’s STEM engagement with loose parts, especially in indoor contexts, remains relatively underexplored^21^. Many studies have focused on the role of outdoor play and physical development^22–26^. While researchers indicate that materials and objects similar to loose parts can enhance children’s cognitive development^21,27^, research is limited on the relationship between children’s cognitive development and their involvement in STEM behaviours. In this study, we observed children’s STEM behaviours during unstructured solitary play with loose parts and with toys that offer limited exploration opportunities (e.g., toy percussion instruments). Our goal was to document cognitive and contextual factors related to early STEM behaviours and engagement. Our findings can inform the design of equitable learning opportunities that align with young children’s backgrounds to scaffold meaningful STEM engagement as building blocks prior to formal instruction or exposure to various other STEM domains (e.g., robotics, coding)^10^.

Research on STEM behaviours in early childhood consistently highlights the role of play-based and exploratory experiences in fostering STEM competencies^2,5,28–30^. STEM in early childhood is a multidimensional domain encompassing distinct disciplines, each grounded in its own theoretical foundations, research base, and pedagogical practices^31,32^. Even very young children spend a significant amount of their time with toys and materials in their environments, leading to forms of discovery learning^33^. Existing studies focus on various STEM experiences, education, and interventions, incorporating a range of technological advancements in early childhood^4,5^. Given young children’s inclination to explore the world around them^34^, observational studies can be critical to understanding the STEM behaviours children engage in and how we can take into account children’s prior knowledge in creating STEM learning environments that are inclusive, effective, and responsive to diverse learning needs^10,35^.

Exploring and manipulating toys and play materials allows young children to formulate scientific foundation serving as potential precursors to learning in STEM subjects^14,36^. However, even seemingly minor characteristics of toys—their quantity, colours, and packaging—can alter children’s play behaviours^37–39^. Construction materials like blocks promote experimentation with balance, symmetry, and mathematical concepts^35,40^, while repurposed items such as cardboard or string may stimulate creativity^41^. Schulz and Bonawitz explored how children’s causal thinking is affected by toys^42^. They found that children collect data by observing, and were more likely to explore toys that offered ambiguous causal relationships than toys that provided expected results. They concluded that this ambiguity creates motivation in children for thoughtful and targeted exploration.

Loose parts (e.g., cardboard tubes, fabric scraps, string, rocks, or containers) can serve as powerful stimuli for STEM exploration and innovation^17,33^. Children independently assess which materials to use based on affordances—the perceived possibilities for action that an object offers^43^. Loose parts can encourage young children to observe properties such as weight, texture, flexibility, and balance, prompting them to ask questions, make predictions, and test outcomes^17^. Children prefer activities that are similar to what adults do with real purpose^44^, and because everyday objects and materials with multiple affordances do not have fixed purposes, they can support open-ended inquiry, encourage problem-solving, and invite children to use trial and error to investigate cause-and-effect relationships. These experiences allow children to transform familiar materials into opportunities for experimentation, reasoning, and scientific thinking in their play^45^.

Research focusing on children’s STEM behaviours with loose parts has been limited. Zeng and Ng conducted a study on unstructured play with loose parts, exploring its power to promote science learning^46^. This research investigated how open-ended questions influence young children’s science learning during play with loose parts in a Singapore kindergarten classroom. They found that open-ended questions extended children’s engagement and increased the complexity of their scientific exploration. Other qualitative studies have similarly focused on children’s STEM learning^19,20,47^. Gull et al. explored how the use of loose parts can address challenges in STEM teaching: their scoping review found 20 studies that emphasize the use of loose parts to encourage creativity, problem-solving, and engineering-like thinking through hands-on engagement with children in elementary classrooms^2^. However, studies with younger children are needed to articulate how play with loose parts may lead to specific STEM behaviours.

While social interactions may enhance STEM learning^12,45,48–50^, solitary play offers an opportunity to observe individual differences in cognitive capacities. In solitary play, without peer or adult scaffolding, children must independently generate ideas, represent problems, plan and test ideas, and evaluate outcomes, engaging multiple cognitive capacities^51^. Play produces cognitive benefits, but cognitive capacities also impact play behaviours^52,53^. Children’s executive function (EF) encompasses core cognitive processes, including working memory, inhibitory control, and cognitive flexibility; EF plays a critical role in children’s ability to engage in complex tasks such as learning and problem-solving^51^.

EF is particularly relevant in play where children must set goals, plan strategies, execute actions, and evaluate outcomes. Exploring children’s EF performance in play is important, as it can predict later achievement in STEM domains, particularly mathematics^54–56^, science^55,57,58^, and engineering^59^. When children interact with toys that limit exploration, their cognitive capacities—particularly EF—may not be fully engaged^60–62^. During solitary play with loose parts, children must hold multiple mental representations, navigate trial and error, and revise strategies^59,63^. To demonstrate how EF and other cognitive capacities serve as a foundation for children’s STEM behaviours and a catalyst for later learning, we must identify the cognitive demands of solitary play with various materials that offer varying degrees of affordances.

Children’s play behaviours arise from multiple contextual influences. Children’s home learning environments, which vary in available educational resources, parental attitudes, and prioritized activities, can be critical determinants of observed play behaviours^64–66^. Researchers find that greater screen exposure can displace active, exploratory play, leading to reductions in both the duration and quality of children’s play^67,68^. Additionally, when parental focus is predominantly directed toward academic tasks, potential opportunities for play may be constrained^69^. However, the home literacy environment—particularly parents’ attitudes toward literacy and numeracy learning—is strongly associated with children’s cognitive outcomes, including science and mathematics achievement^64,70,71^. Research is warranted on how the home environment may support STEM-related learning versus other activities, as these home learning opportunities and parental priorities can shape the thematic direction of children’s play^72,73^. Examining children’s learning trajectories is important for STEM education and supports progressively complex skill development through various stages^74^.

Children’s engagement with STEM is also shaped by the social context of play. Gendered socialization, including the marketing and packaging of toys, influences children’s interest in and access to STEM activities. Studies show that subtle cues such as labelling a mechanical toy as for boys or girls can alter both children’s behaviours and parental involvement in play^37^. This gendered patterning early in life can affect self-efficacy, interest, and persistence in STEM^75,76^. Understanding interactions between cognitive and contextual factors becomes essential for identifying the mechanisms that support children’s STEM interests and competencies.

This study addresses two key gaps in the literature on early childhood STEM learning. First, this work investigates how play materials like loose parts shape children’s spontaneous STEM behaviours, and how these behaviours may be related to children’s cognitive capacities—particularly their EF. Although research has examined how early STEM exposure supports cognitive development (e.g., Gold et al.), much of this work has focused on social contexts^77^. Far less attention has been given to how children engage in STEM behaviours during solitary play, despite its relevance for understanding individual cognitive processes.

Second, this study explores how home learning environment and parental attitudes towards play contribute to children’s play behaviours^78–80^. The differences in home learning environments and parental attitudes may also shift the development of young children’s self-regulatory capacities—EF, attentional control, and behavioural regulation skills—which strongly predict children’s play behaviours and school readiness^81–83^.

The present study examined how material type (loose parts vs. limited-purpose toys) influenced the frequency and type of STEM behaviours during solitary play. We also investigated how individual and contextual factors relate to variation in children’s STEM behaviours and overall engagement. The study was guided by the following research questions:

1. What types of STEM behaviours do children exhibit when playing with loose parts compared to toys that allow limited opportunities for exploration?
2. What are the predictors of children’s STEM behaviours and engagement with loose parts?

We predicted that children would engage in more STEM behaviours with loose parts than with toy percussion instruments (the control condition), and that children’s cognitive capacities—particularly their executive function—would influence their overall engagement.

## Methods

There was no preregistration for this study.

### Participants

Children and their parents were recruited as participants from private and not-for-profit daycares in a large city in western Canada between July 2022 and May 2024. Participants in this study were not compensated. In our data analysis, we included 60 children who participated in two play sessions, completed all cognitive assessments, and had parents who completed a parental questionnaire. Table 1 includes the characteristics of our participants, which were gathered through parent questionnaires. We asked parents to report on their children’s sex (i.e., male, female), and provided them with the option to self-describe, if they wanted. Most parents identified as mothers (83%),were born in Canada (83%), and reported their child was also born in Canada (95%). We did not collect information on race or ethnicity. Monolingual children accounted for 47% of the sample, while children who may be exposed to more than one language at home accounted for 53%. Parental education was measured on a 6-point scale, from 1 = less than high school to 6 = graduate degree. The median level of parental education was 5.00 (university graduate; interquartile range [*IQR*] = 1.00). The quantity of books in the home was measured on a 6-point scale, from 1 = 0–25, to 6 = 200 or more. The median number of children’s books at home was 4.00 (76 to 100 books, *IQR* = 3.00). The median number of adult books at home was 4.00 (76 to 100 books, *IQR* = 2.50). Reading to children was measured on a 9-point scale, from 1 = never, to 9 = more than 7 times a week. The median number of readings that occurred per week at bedtime was 8.00 (7 times a week, *IQR* = 1.00) and 5.00 at other times (4 times a week, *IQR* = 2.00).

**Table 1:** Participant characteristics

Characteristic	*n* (%)
Sex (*n*, % of total sample)	
Male	28 (46.7%)
Female	32 (53.3%)
Parent answered questionnaire	
Mother	50 (83.3%)
Father	9 (15.0%)
Other	1 (1.7%)
Home language	
Monolingual	28 (46.7%)
Multilingual	32 (53.3%)
Children’s birth country	
Canada	57 (95.0%)
Other	3 (5.0%)
Parents’ birth country	
Canada	50 (83.3%)
Other	10 (16.7%)

### Ethical considerations

Parents and caregivers provided written informed consent, and the children gave verbal assent to participate in the study. The study was approved for the procedures by the MacEwan University Research Ethics Board (File No: 101952).

## Measures

### Cognitive assessments

Given the potential influence of cognitive development on children’s play behaviours, two assessments were employed to evaluate cognitive capacities: the Wechsler Preschool and Primary Scale of Intelligence, Fourth Edition: Canadian (WPPSI-IV)^84^, and the Head–Toes–Knees–Shoulders Task (HTKS Task)^85^, which assesses EF performance. The sequence of the HTKS Task and the WPPSI-IV administration was randomized.

### The WPPSI-IV

This standardized assessment is designed to evaluate children’s cognitive abilities (age 2:6 to 7:7). The assessment includes 15 subtests organized into cognitive domains. Raw scores were first converted into scaled scores and used for creating composite scores used in the analysis as follows: Verbal Comprehension Index (VCI) assesses verbal reasoning and language comprehension; Visual Spatial Index (VSI) evaluates visual perception and spatial problem- solving; Fluid Reasoning Index (FRI) measures logical thinking and problem-solving with novel information; Working Memory Index (WMI) examines short-term memory and manipulation of visual or spatial information; and Processing Speed Index (PSI) assesses the speed and accuracy of visual information processing. Full-Scale IQ (FSIQ), derived from five subtests for younger and six for older children, provides a comprehensive measure of overall cognitive functioning.

### HTKS task

The HTKS task measures EF performance in young children, particularly cognitive flexibility, working memory, and inhibitory control^86,87^. It involves behavioural regulation through structured instructions. The task required children to perform actions opposite to verbal instructions they received, challenging their ability to suppress automatic responses and apply rule-based behaviours. Children received 0 for incorrect responses (e.g., touching the prompted body part, such as “head”), 1 for self-corrected responses, and 2 for correct responses (e.g., touching the opposite body part, such as “toes”). The measure was scored on a scale of 1–62. Task duration varied depending on the child’s performance and ability to progress through the stages.

### Parent questionnaire

The parent questionnaire was designed to collect information on children’s play experiences, home environments, and parental perspectives. The quality of the home learning environment plays a critical role in children’s development^64,88–92^.

The first section of the parent questionnaire gathered detailed demographic and socioeconomic data about the child and the parent. Parents provided information on their child’s sex, date of birth, country of birth, as well as their relationship to the child, country of birth, and postal code. Additionally, they reported their highest level of education and language use at home. The questionnaire also included items on the number of books in the home and how often parents or household members read to the child each week, distinguishing between bedtime reading and other reading times. The questionnaire also assessed parents’ attitudes toward early childhood literacy, math, science, screen time, and play, using a four-point Likert scale from “strongly agree” to “strongly disagree.” Parents also reported how frequently their child engaged in various activities and how often they participated together, including math (e.g., counting games), reading (e.g., pointing to letters), and creative play (e.g., building, pretend play), using a five-point scale from “never” to “always.” These items were adapted from prior studies on parental beliefs and practices related to children’s education and development^93,94^.

### Play materials and toys

**Fig. 1:**
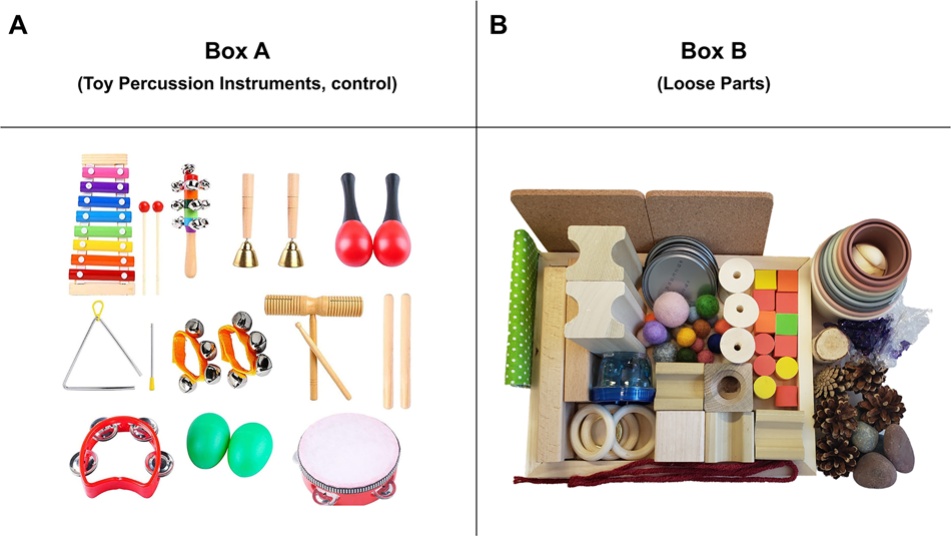
Play session items. Panel **A** shows the content of Box A with toy percussion instruments. Panel **B** shows the content of Box B with loose parts.

The play session materials for this study were organized into two distinct sets, labelled Box A and Box B. The toy boxes used in this study were 12.9-quart clear plastic, providing a uniform and secure storage solution for the materials in Box A (toy percussion instruments, control) and Box B (loose parts). See Fig. 1 below for the contents of Boxes A and B. For Box A, the control condition, we selected toy percussion instruments because, while they are multi-piece and varied in texture like loose parts, they offer limited affordances for play. The second set, Box B, consisted of a diverse range of loose parts, which were selected to be gender-neutral and free of explicit play cues. To ensure consistency, the box contents were presented to children in a standardized arrangement. Similarly, the percussion instruments were a varied set, rather than a standalone toy, ensuring that both conditions offered diverse interaction opportunities while differing in their affordances and constraints.

### Procedures

### Play sessions

Children participated in two play sessions, each lasting up to 30 minutes. They were randomly assigned to one of the conditions first: playing with toy percussion instruments or with loose parts. Children played with the alternative box in the next play session, at least two days after the first session. In the session’s final minutes, or if the child indicated they were done, the researcher asked the child what they were doing. This approach encouraged children to reflect on and explain their play while preserving the natural flow of interaction.

### Cognitive assessment sessions

In Session 3, the researcher administered the WPPSI-IV and the HTKS task in a quiet, distraction-free room. The assessments were conducted individually, following standardized procedures. WPPSI-IV was administered in a standardized order, tailored to the child’s age group. The assessment was split into two shorter sessions if the child needed a break.

## Observational data coding

### Play duration

The play sessions were observed and coded on a minute-by-minute basis^94,95^. This coding process involved identifying when children’s play started and ended. In rare cases, if children took a break (e.g., used the bathroom), the researcher stopped, resumed the time once the child was back, and recorded the minutes of play.

### STEM behaviours

The observed STEM behaviours were coded using the STEM Play Behaviour Scale, which consists of eleven subtypes, outlined in Table 2. This scale was adapted from previous research focusing on children’s STEM and engineering play behaviours^41,45,95,96^. Researchers watched video recordings of each play session and coded children’s STEM behaviours. Children were assigned either 1 or 0 for each minute across all STEM behaviours, indicating whether the behaviours occurred or did not occur during the one-minute time frame. Some of the STEM behaviours required a verbal component, such as ‘communicating goals,’ whereas some required a non-verbal component (e.g., constructing structures). Higher quality play could feature multiple STEM behaviours per minute. A sum for each behaviour was calculated for the play session and then divided by the duration to create a ratio. For instance, if a child’s play session lasted 10 min and they engaged in a specific STEM behaviour during 5 of the 10 minutes, the child’s frequency for that exploration would be 0.5 (i.e., 50%). This adjustment ensured that the resulting frequencies reflected the prevalence and distribution of STEM behaviours within each play session. A STEM Engagement Score was calculated by summing all observed STEM behaviours.

**Table 2:** STEM behaviour coding descriptors

STEM behaviours	Description
Evaluating design	Test the function of a completed design.
Following prototypes	Compare how something looks in the real world and recreate it with their materials.
Integrating technological ideas	Incorporate elements of technology where there is no technology (e.g., “The machine will pull this up”).
Using STEM-specific language	Use language specific to the field of STEM (e.g., ramp, gravity, stability).
Testing hypotheses	An element of curiosity while redesigning a constructed item.
Explaining how things are built or work	Describe their creation(s) either during or after construction.
Constructing structures	Gather, sort, or stack materials to create a structure or design.
Exploring mathematical concepts	Involve spatial reasoning, pattern recognition, or common mathematical domains.
Solving problems	Propose solutions to challenges.
Communicating goals	State their objectives and plans.
Asking questions	Pose questions to gather information about the function of an object or materials.

### Interrater reliability assessment

An interrater reliability assessment was conducted to ensure the reliability of the coding process. A team of four researchers coded the data. During the initial training, our research team observed play sessions together, discussing and categorizing STEM behaviours within each category. Researchers independently coded STEM behaviours and then compared the results.

Discrepancies were resolved through discussion with the first author. Interrater reliability was assessed using a subset of 13 randomly selected participant sessions (21% of the total 60 sessions). A subset was chosen due to the time-intensive nature of video coding. While conducting reliability analysis on the full dataset would offer the most comprehensive check, randomly selecting a portion is a widely accepted practice in observational research^97^. Each selected play session was independently coded by multiple researchers, and the resulting codes were compared to evaluate consistency. Following Tong et al., intraclass correlation coefficients (ICCs) were used as they were appropriate for assessing interrater reliability with ratio-level data and multiple raters^98^. ICCs were specifically calculated for the STEM Engagement Score to determine agreement across coders. Based on the guidelines procured by Koo and Li, the ICC was calculated using a one-way random-effects model^99^. For the STEM Engagement Score, the single measure ICC was 0.914 (*F*([623, 624)] = 22.4, *p* < 0.001, 95% CI [0.901, 0.926]), indicating excellent interrater reliability. ICCs were not correlated to the child’s gender, age, or multilingualism.

### Statistics

Statistical analysis was conducted using SPSS Statistics (Version 29.0.2.0)^100^ and JASP (Version 0.19.3.0)^101^. A significance level of α = 0.05 was used unless stated otherwise. Data were summarized using medians, interquartile ranges (IQRs), and ranges (min–max). Spearman’s correlation coefficients were reported for all numerical measures. Wilcoxon signed-rank tests were used to compare the median frequencies of STEM behaviours between the two conditions (loose parts and toy percussion instruments), as these measures were not normally distributed.

An exploratory factor analysis was conducted to produce composite scores and summarize the results from the parental questionnaire. The identified composite scores and other measurements were included in a forward selection linear regression to determine which factors predicted children’s STEM behaviours with loose parts. Using forward selection helps avoid multi-collinearity and issues of overfitting while identifying the highest impact predictors of the STEM Engagement Score^102^. Entering all predictors simultaneously could have led to overfitting, particularly when some predictors (e.g., cognitive functioning, EF performance, age) were highly correlated, making the model unreliable. Multicollinearity may have reduced the precision of the estimated coefficients, potentially affecting the model’s interpretability.

Forward regression allowed us to mitigate these risks by selecting the most important predictors step by step. We added each predictor with the strongest relationship to the outcome and then sequentially included other predictors that continued to improve the model. This process was repeated until no additional variables significantly enhanced the model. For the Wilcoxon signed-rank test analysis, a Bonferroni correction was applied to the significance level to account for multiple comparisons across different measures of children’s STEM behaviours. In this case, we performed 13 tests, leading to a Bonferroni-adjusted significance level of 0.0038, or 0.38%. This conservative adjustment ensured that the significant results reported were less likely to be due to random chance and more likely to reflect true differences.

## Results

### Descriptive statistics

Table 3 below presents the medians, IQR, and range of scores for key variables in the study.

**Table 3:** Medians and IQR of key variables

Key variables	*n*	*Mdn*	*IQR*	Min–Max
Verbal Comprehension Index (VCI)	60	105.0	18.8	58–141
Visual Spatial Index (VSI)	60	107.5	31.0	65–145
Fluid Reasoning Index (FRI)	52	97.0	17.8	58–127
Working Memory Index (WMI)	60	103.0	19.0	45–129
Processing Speed Index (PSI)	52	100.0	15.8	66–123
Full-Scale IQ (FSIQ)	60	131.5	47.5	57–170
EF Performance (HTSK task)	60	51.0	25.0	0–62
Play Duration (control)	60	26.0	10.0	10–30
Play Duration (loose parts)	60	30.0	4.0	5–30
STEM Engagement Score (control)	60	0.2	0.4	0.0–1.5
STEM Engagement Score (loose parts)	60	1.3	0.7	0.0–2.9

FRI and PSI have lower *n* due to the age criteria for administering these measures.

### Correlational analyses

The Spearman correlation analysis indicated several significant relationships among cognitive functioning, EF, and children’s STEM behaviours with loose parts and toy percussion instruments (see Table 4). Children’s age was positively correlated with cognitive functioning (FSIQ), *r*(58) = 0.57, *p* < 0.001, Fisher’s *z* = 0.65, 95% *CI* [0.37, 0.72] and EF performance (HTKS), *r*(58) = 0.65, *p* < 0.001, Fisher’s *z* = 0.77, 95% *CI* [0.47, 0.77]. FSIQ was significantly correlated with all its subscales and EF Performance, *r*(58) = 0.53, *p* < 0.001, Fisher’s *z* = 0.60, 95% *CI* [0.32, 0.69]. STEM Engagement Score in the loose parts condition was significantly correlated with FSIQ, *r*(58) = 0.46, *p* < 0.001, Fisher’s *z* = 0.49, 95% *CI* [0.23, 0.64], and with total STEM Engagement Score in the control condition, (58) = 0.51, *p* < 0.001, Fisher’s *z* = 0.56, 95% *CI* [0.29, 0.68].

**Table 4:** Spearman correlations between variables

Variables	1	2	3	4	5	6	7	8	9	10	11	12
1. Age (in months)	–											
*n*	–											
2. Parent education	−0.041	–										
*n*	60	–										
3. Verbal Comprehension Index	0.180	−0.010	–									
*n*	60	60	–									
4. Visual Spatial Index	0.178	−0.152	0.246	–								
*n*	60	60	60	–								
5. Fluid Reasoning Index	−0.025	0.020	0.308*	0.474***	–							
*n*	52	52	52	52	–							
6. Working Memory Index	0.032	−0.107	0.304*	0.396**	0.296*	–						
*n*	60	60	60	60	52	–						
7. Processing Speed Index	0.155	0.019	0.397**	0.294*	0.353*	0.389**	–					
*n*	52	52	52	52	52	52	–					
8. Full-Scale IQ (FSIQ)	0.570***	−0.233	0.379**	0.600***	0.369**	0.293*	0.437**	–				
*n*	60	60	60	60	52	60	52	–				
9. EF Performance	0.646***	0.115	0.280*	0.147	0.166	0.142	0.345*	0.533***	–			
*n*	60	60	60	60	52	60	52	60	–			
10. Play Duration (control)	0.185	0.190	0.159	−0.017	−0.043	−0.013	0.049	0.152	0.290*	–		
*n*	60	60	60	60	52	60	52	60	60	–		
11. Play Duration (loose parts)	0.084	0.066	0.100	0.123	0.141	−0.044	−0.239	0.131	0.090	0.392**	–	
*n*	60	60	60	60	52	60	52	60	60	60	–	
12. STEM Engagement (control)	0.110	0.110	0.228	0.100	−0.105	−0.115	0.093	0.187	0.024	0.212	−0.032	–
*n*	60	60	60	60	52	60	52	60	60	60	60	–
13. STEM Engagement (loose parts)	0.112	0.021	0.180	0.071	0.078	−0.143	−0.020	0.271*	0.139	0.094	0.146	0.492***
n	60	60	60	60	52	60	52	60	60	60	60	60

* *p* < 0.05; ** *p* < 0.01; *** *p* < 0.001.

### Examining sex differences in cognitive development and STEM engagement

A series of multivariate analyses of variance (MANOVAs) was conducted to examine whether children’s sex (*N*_male_ = 28, *N*_female_ = 32) influenced their cognitive functioning (FSIQ), EF performance, and STEM Engagement Score across play conditions. The MANOVAs were not significant (all *p*-values > 0.05). To examine the null findings, we fit a Bayesian ANOVA for each outcome, comparing models with and without sex (see Table 5). Across EF performance and STEM engagement scores in both play conditions, Bayes factors favoured the null (BF₀₁ = 3.41–3.79; equivalently, BF₁₀ = 0.26–0.29). Assuming equal prior model odds, the posterior probability of the null ranged from 0.78 to 0.79 (77.4–79.1%), constituting moderate evidence for no sex effect on EF performance or STEM engagement score. In contrast, evidence for or against an effect of sex on FSIQ was inconclusive. Bayesian model comparison indicated that the data were slightly more likely under the null model than under the sex-difference model (BF₀₁ = 1.25; BF₁₀ = 0.80), providing only weak evidence in favour of the null; thus, we found no convincing evidence for or against the effect of sex on cognitive functioning, but moderate evidence for no effect of sex on EF performance, or STEM Engagement Score.

**Table 5:** Summary of Bayesian ANOVA results comparing models with and without sex across outcomes

	BF₁₀	BF₀₁	P(M | data)	*n*_male_/*n*_female_	Mean ± SD (M)	Mean ± SD (F)	Interpretation
FSIQ	1.00	0.80	0.445	32/28	135.7 ± 26.6	121.9 ± 28.5	Weak evidence for null; no convincing sex effect
HTKS	0.29	3.43	0.774	32/28	42.6 ± 22.3	39.9 ± 20.8	Moderate evidence for no credible sex effect
STEM engagement (control)	0.27	3.66	0.785	32/28	0.37 ± 0.40	0.40 ± 0.40	Moderate evidence for no credible sex effect
STEM engagement (loose parts)	0.26	3.79	0.791	32/28	1.34 ± 0.54	1.35 ± 0.64	Moderate evidence for no credible sex effect

Equal prior model odds assumed (P(M) = 0.50).

### Differences in children’s STEM behaviours and engagement in play

A series of Wilcoxon signed-rank tests was conducted to examine differences in children’s STEM behaviours between the loose parts and toy percussion instrument (control) conditions. A Bonferroni correction was applied to control for Type I error across 13 comparisons, establishing a significance threshold of *p* < 0.0038. Effect sizes are reported as matched rank biserial correlations (*d*), with corresponding standard errors and 95% confidence intervals. Table 6 shows the results for STEM behaviours and Engagement Scores. We also included the duration of each condition for benchmarking children’s play length.

**Table 6:** Paired samples Wilcoxon signed-rank test results for STEM behaviours, engagement, and play session duration

	Statistic s (*U*)	*z*	*df*	*p*	Effect size	SE Effect size	Lower	Upper
Evaluating design	43.50	2.49	59	0.015	0.93	0.36	0.74	0.98
Following prototypes	239.50	0.83	59	0.412	0.18	0.21	−0.24	0.54
Integrating technological ideas	36.00	1.60	59	0.112	0.60	0.36	−0.04	0.89
Using STEM- specific language	31.00	0.36	59	0.751	0.13	0.34	−0.52	0.68
Testing hypotheses	137.00	2.24	59	0.026	0.60	0.26	0.17	0.84
Explaining how things are built/work	1188.50	5.32	59	<0.001*	0.86	0.16	0.76	0.93
Exploring mathematical concepts	969.00	5.10	59	<0.001*	0.87	0.17	0.77	0.93
Constructing structures	1711.00	6.62	59	<0.001*	1.00	0.15	1.00	1.00
Solving problems	141.50	0.90	59	0.373	0.23	0.24	−0.25	0.62
Communicating goals	724.00	3.80	59	<0.001*	0.68	0.18	0.45	0.83
Asking questions	394.00	−1.80	59	0.072	−0.30	0.17	−0.57	0.02
STEM engagement score	1770.00	6.68	59	<0.001*	1.00	0.15	1.00	1.00
Play session duration	734.50	3.54	59	<0.001*	0.63	0.18	0.37	0.79

The effect size is reported as the matched rank biserial correlation. Results are considered significant only if the *p*-value is below 0.05/13 (i.e., 0.0038) and marked with a * sign if they are significant.

**Fig. 2:**
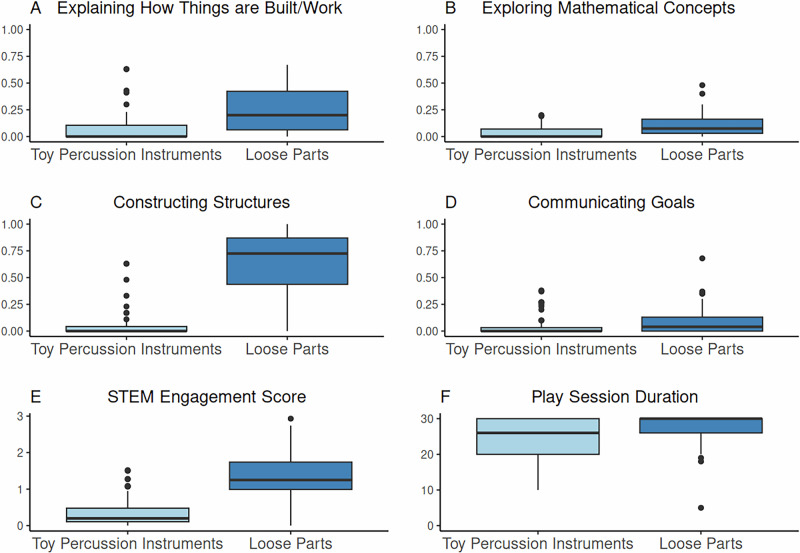
Boxplots comparing children’s play behaviours, engagement, and play session duration with toy percussion instruments (control) and loose parts*.* Panels **A**–**D** show children’s STEM behaviours for the control and loose parts sessions; only STEM behaviour variables that differed significantly between conditions are included. Panels **E** and **F** show children’s STEM Engagement Scores and play session duration for the control and loose parts sessions. Each box represents the middle 50% of measurements, whiskers indicate the lowest and highest 25%, and dots denote outliers. *N* = 60.

Children explained how things were built or worked, explored mathematical ideas, constructed structures, and communicated their goals more frequently in the loose parts condition compared to the control. Overall, their total STEM Engagement Score was higher during play with loose parts, and they also spent more time playing with loose parts compared to the toys in the control condition. See Fig. 2 for significant results for four STEM behaviours, STEM Engagement Score, and Play Duration between conditions.

### Differences in STEM behaviours within each condition

Two repeated-measures one-way ANOVAs were conducted to examine differences in the mean proportion of time children engaged in each of the STEM behaviours. Post-hoc pairwise comparisons with Bonferroni correction were conducted to identify differences between specific behaviours. With loose parts, there was a significant effect of STEM behaviour type on the mean proportion of time spent on the different behaviours, *F*(10, 590) = 146.60, *p* < 0.001, ηp² = 0.71. Post-hoc analysis indicated that “constructing structures” occupied a significantly greater portion of time than all other STEM behaviours (*Mdiff* = 0.422-0.661, *SE*s = 0.033–0.036, *p*s < 0.001, *d*s = 3.28–5.14). The second most frequent STEM behaviour was “explaining how things work,” which was significantly more frequent than “testing hypotheses” (*Mdiff* = 0.202, *SE* = 0.029, *p* < 0.001, *d* = 1.57), “using STEM language” (*Mdiff* = 0.234, *SE* = 0.026, *p* < 0.001, *d* = 1.82), “integrating technology” (*Mdiff* = 0.232, *SE* = 0.026, *p* < 0.001, *d* = 1.80), and “evaluating design” (*Mdiff* = 0.239, *SE* = 0.026, *p* < 0.001, *d* = 1.86). The third most frequent STEM behaviour was “exploring math concepts,” and was significantly greater than “testing hypotheses” (*Mdiff* = 0.066, *SE* = 0.019, *p* < 0.043, *d* = 0.51), “using STEM language” (*Mdiff* = 0.098, *SE* = 0.013, *p* < 0.001, *d* = 0.77), “integrating technology” (*Mdiff* = 0.096, *SE* = 0.013, *p* < 0.001, *d* = 0.75), and “evaluating design” (*Mdiff* = 0.103, *SE* = 0.013, *p* < 0.001, *d* = 0.80).

**Fig. 3:**
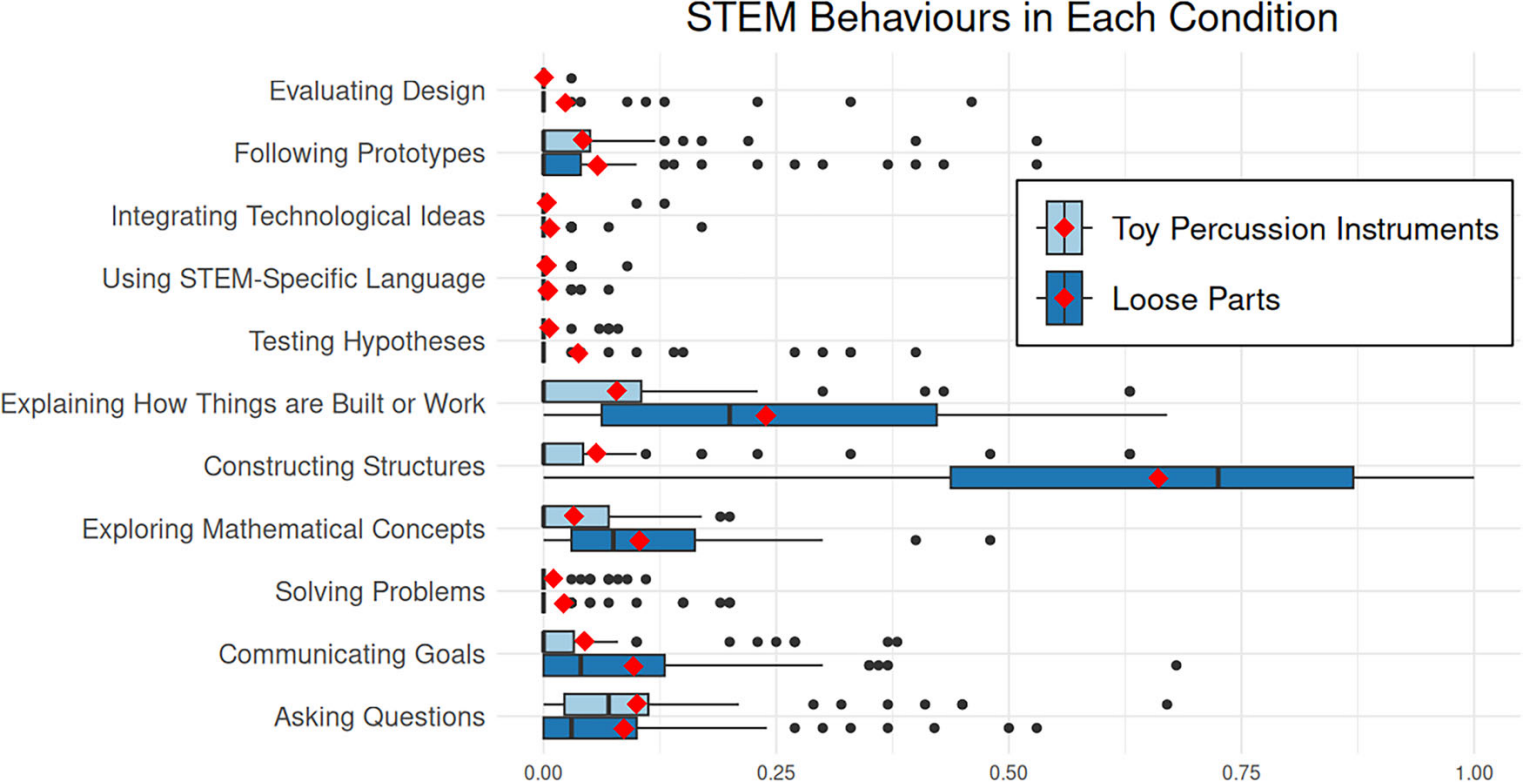
Proportions of time for STEM behaviours with toy percussion instruments (control) and loose parts. Proportions of each STEM behaviour in the toy percussion instrument (control) and the loose parts sessions. The mean proportion of each STEM behaviour is marked with a red diamond. The light blue boxes represent the data collected in the Toy Percussion condition, and the dark blue boxes represent the data in the Loose Part condition. *N* = 60.

With toy percussion instruments, there was a significant effect of STEM behaviour type on the mean proportion of time spent on the different behaviours, *F*(10, 590) = 10.54, *p* < 0.001, ηp² = 0.15. Post-hoc analysis indicated that “asking questions” occupied a significantly greater portion of time than “communicating goals” (*Mdiff* = 0.056, *SE* = 0.015, *p* = 0.019, *d* = 0.66), “exploring math concepts” (*Mdiff* = 0.067, *SE* = 0.018, *p* = 0.020, *d* = 0.78), “solving problems” (*Mdiff* = 0.089, *SE* = 0.017, *p* < 0.001, *d* = 1.06), “testing hypotheses” (*Mdiff* = 0.094, *SE* = 0.018, *p* < 0.001, *d* = 1.11), “integrating technology” (*Mdiff* = 0.096, *SE* = 0.018, *p* < 0.001, *d* = 1.14), “using STEM language” (*Mdiff* = 0.097, *SE* = 0.017, *p* < 0.001, *d* = 1.14), and “evaluating design” (*Mdiff* = 0.100, *SE* = 0.017, *p* < 0.001, *d* = 1.18). The second most frequent STEM behaviour was “explaining how things work,” which occupied significantly more time than “testing hypotheses” (*Mdiff* = 0.072, *SE* = 0.018, *p* = 0.013, *d* = 0.85), “using STEM language” (*Mdiff* = 0.075, *SE* = 0.018, *p* = 0.005, *d* = 0.89), “integrating technology” (*Mdiff* = 0.075, *SE* = 0.017, *p* = 0.003, *d* = 0.88), and “evaluating design” (*Mdiff* = 0.078, *SE* = 0.018, *p* = 0.004, *d* = 0.92). The third most frequent STEM behaviour was “constructing structures,” which was not significantly different from any other STEM behaviour. Two graphs were created to examine the percentage of children’s play that involves each STEM behaviour (see Fig. 3).

## Regressions

### Exploratory factor analysis and composite score development

Our exploratory factor analysis identified six factors explaining 46.7% of the variance in the parental questionnaire data: Frequency of Home Learning Activities (Factor 1), Parental STEM Attitudes (Factor 2), Frequency of Home Numeracy Activities (Factor 3), Parental Play Attitudes and Engagement (Factor 4), Home Literacy Environment (Factor 5), Parental Literacy Attitudes (Factor 6). The composite scores for these factors were computed and used for further analysis (For Details, see Supplementary Information, Supplementary Note 1, and Table 1). Predictors and potential covariates of STEM behaviours were examined using linear regressions. These covariates included the child’s age in months, sex, parental education^64^, cognitive skills measured by various composite scores (i.e., WPPSI-IV’s VCI, VSI, FRI, WMI, PSI, and FSIQ), and EF performance (i.e., HTKS Task).

Our within-subjects design allowed each child to experience both conditions; the order of the play conditions was randomly assigned. The play session order was also tested as a covariate to determine whether the sequence in which children were exposed to materials affected their STEM behaviours due to effects such as fatigue, increased familiarity, or a preference for the materials presented earlier. This approach ensured that observed differences in STEM behaviours can be attributed to the materials or activities rather than other factors.

### Factors predicting STEM engagement

A linear regression analysis with forward selection was conducted to examine predictors of children’s STEM Engagement Score while using toy percussion instruments (control). The final model accounted for 8.1% of the variance (*R*² = 0.08, adjusted *R*² = 0.06, RMSE = 0.373) and was statistically significant, *F*(1, 50) = 4.42, *p* = 0.040. VCI predicted STEM Engagement Score, *B* = 0.007, *SE* = 0.003, *β* = 0.29, *t*(50) = 2.10, *p* = 0.040. The intercept was not statistically significant (*B* = −0.36, *SE* = 0.35, *t*(50) = −1.03, *p* = 0.308). Covariates tested but excluded from the final model were child age, sex, parental education, play session order, home learning environment factors, EF performance, and other cognitive functioning composite scores (VSI, FRI, WMI, PSI, and FSIQ).

**Fig. 4:**
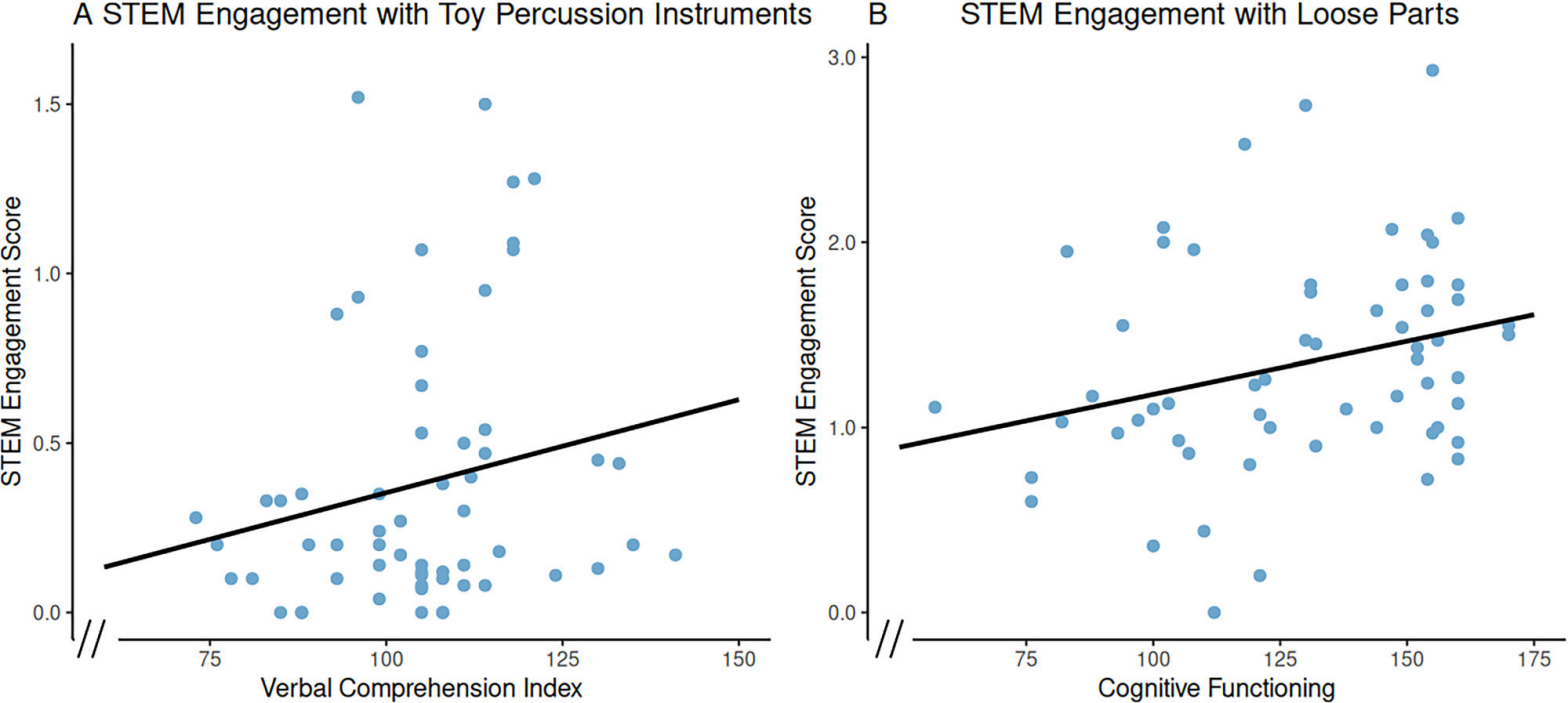
Relationships between significant predictors of STEM engagement scores. Panel **A** indicates the relationship between children’s STEM Engagement Scores in the control condition and the predictor VCI. Panel **B** indicates the relationship between children’s STEM Engagement Score in the loose parts condition and the predictor cognitive functioning. *N* = 52.

Another linear regression analysis with forward selection was conducted to examine predictors of children’s STEM Engagement Score in the loose parts condition. The final model explained 15.2% of the variance (*R*² = 0.15, adjusted *R*² = 0.14, RMSE = 0.522) and was statistically significant, *F*(1, 50) = 8.95, *p* = 0.004. Cognitive functioning (FSIQ) emerged as a significant predictor, *B* = 0.008, *SE* = 0.003, *β* = 0.39, *t*(50) = 2.99, *p* = 0.004, indicating that higher overall cognitive functioning (FSIQ) is positively associated with higher STEM Engagement Score with loose parts. The intercept was not statistically significant (*B* = 0.28, *SE* = 0.36, *t*(50) = 0.78, *p* = 0.437), indicating that variation in STEM Engagement Score was largely attributed to differences in cognitive functioning (FSIQ). Covariates tested but excluded from the final model were child age, sex, parental education, play session order, home learning environment factors, EF performance, and other cognitive functioning composite scores (VCI, VSI, FRI, WMI, and PSI). FRI and PSI have lower *n* due to the age criteria for administering these measures. See Fig. 4 for the relationship between significant predictors and STEM engagement.

An additional set of linear forward selection regressions was conducted to examine predictors of children’s STEM Engagement Score when constructing structures were excluded. This analysis aimed to determine whether the other 10 STEM behaviours—which may rely more on children’s verbal capacities, in contrast to mostly action-based and non-verbal construction behaviours—would be predicted by a different set of factors. The predictors have not changed (for details, see Supplementary Information for this additional analysis as Supplementary Note 2 and Fig. 1).

### Factors predicting the construction behaviours with loose parts

**Fig. 5:**
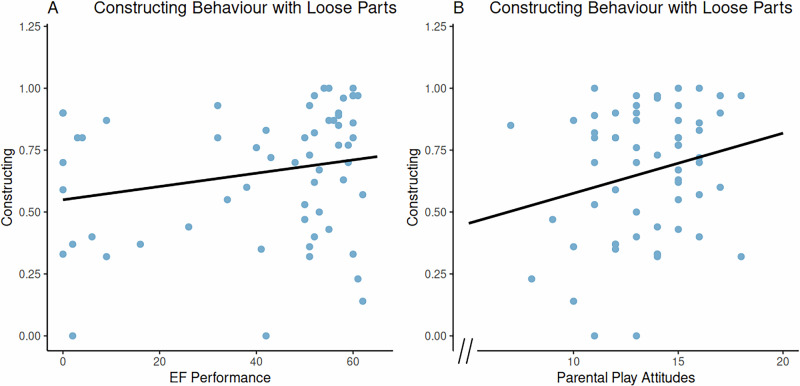
Relationships between significant predictors of constructing structures with loose parts. Panel **A** displays the relationship between children’s STEM behaviour of constructing structures in the loose parts condition and the predictor EF performance. Panel **B** displays the relationship between children’s STEM behaviour of constructing structures in the loose parts condition and the predictor parental play attitudes. *N* = 52.

In our study, children engaged significantly more in constructing structures—more than any other STEM behaviours—during play with loose parts compared to the control condition. In addition, most other STEM behaviours we examined in solitary play relied primarily on children verbalizing their ideas, whereas constructing structures could be assessed both verbally and non- verbally. We therefore examined the factors associated with increased involvement in constructing behaviours alone. To identify predictors of children’s construction with loose parts, we conducted a linear regression analysis using a forward selection approach. We specifically aimed to determine whether the composite scores from the WPPSI-IV (VCI, VSI, FRI, WMI, PSI, and FSIQ) would uniquely predict children’s construction behaviours with loose parts. The final model was statistically significant, *F*(2, 49) = 6.73, *p* = 0.003, and explained 21.6% of the variance in children’s constructing behaviours, *R²* = 0.22, Adjusted *R²* = 0.18. Both EF performance (*β* = 0.35, *t* = 2.72, *p* = 0.009) and parental play attitudes and engagement (Factor 4; *β* = 0.27, *t* = 2.08, *p* = 0.043) were significant predictors. Children with strong EF performance and those from families with highly positive play attitudes and frequent play engagement constructed most frequently with loose parts. The following covariates were considered during model selection, but were not retained due to non-significant contributions: child’s age, sex, parental education, play session order, home learning environment factors, and cognitive functioning composite scores (VCI, VSI, WMI, FRI, and FSIQ). See Fig. 5 for the relationship between significant predictors and constructing structures.

## Discussion

Using a within-subjects design, this study compared children’s STEM behaviours and engagement during unstructured solitary play with two types of materials: versatile objects (i.e., loose parts) and toy percussion instruments (control).

### Differences in children’s STEM behaviours and engagement in play

The Wilcoxon signed-rank tests demonstrated that children engaged in significantly more STEM behaviours when playing with loose parts than with toy percussion instruments, particularly in constructing structures, communicating goals, explaining how things are built or work, and exploring concepts related to math. These behaviours not only occurred more frequently, but also with large effect sizes, suggesting the play material affordances of loose parts facilitate more diverse and complex STEM engagement.

In line with the suggestions of researchers who have explored young children’s STEM innovation, thinking, and learning^4,46,77^, our study provided experimental and observational evidence of the connection between a sizable sample of children’s indoor STEM behaviours and engagement with a variety of loose parts. Despite the variability across individual subcategories, the overall proportion of STEM behaviours and engagement was significantly higher in the loose parts condition. These findings extend qualitative work that suggests that loose parts may lead to STEM learning opportunities^17,103^.

Our categories of children’s STEM behaviours were in line with previous research^41,45,95,96^, and our work provides a comprehensive picture of what children spontaneously do by themselves with a variety of loose parts. For example, Gold and colleagues showed that, compared to traditional outdoor playgrounds and indoor dramatic play areas, children demonstrated significantly higher frequencies of design and construction behaviours when playing with these materials^77^. While our coding schema captured a broader range of STEM behaviours than theirs, consistent with these earlier findings, we observed that children engaged more frequently in STEM behaviours when using loose parts compared to toy percussion instruments.

While Gold et al. primarily focused on engineering behaviours, other researchers have examined how STEM learning may emerge during different types of play^77^. For example, Thibodeau-Nielsen et al. found that children produced STEM-related language only 16% of the time during solitary play, which was the second lowest rate among play types they explored (i.e., play involving peers)^45^. In contrast, our study focused exclusively on solitary play with younger children and used a broader coding scheme that captured both verbal and non- verbal STEM behaviours (e.g., constructing). Our approach provided a more wide-ranging account of children’s engagement with STEM concepts. Most STEM behaviour categories in our coding framework captured children’s verbal descriptions during play. However, the largest proportion of non-verbal observations occurred in the categories of constructing structures and exploring mathematical concepts, where children’s actions rather than speech were the primary indicators of behaviour.

### Do different play materials and toys lead to differentiated STEM behaviours?

We found that materials with many affordances support more frequent STEM behaviours and overall engagement. Our within-subjects design strengthens this interpretation, indicating that the increase in STEM behaviours is attributable to the material and toy context rather than variability across children. Children’s play materials shape their play behaviours and exploration of concepts such as science, math, problem-solving, goal setting, and planning^42,104,105^, and different types of toys and materials can stimulate distinct domains of development. For instance, toys that promote creative construction and social fantasy encourage imagination and innovation in children, helping them build complex play scenarios^61^. Similarly, the nature of toys directly affects how children engage in play and learning^106^. These findings emphasize that materials and toys are active tools that guide how children think, imagine, and relate to the world.

In our study, children were predominantly engaged in constructing structures. Other studies classify STEM behaviours in various ways as play (e.g., constructive, engineering, or loose parts play)^2,59,107^. Construction with objects is a prominent part of children’s lives in early childhood^14,108^. Yet researchers debate whether construction behaviours constitute play^22,108^ or not^14,109^. The researchers who do not consider construction as play explain that—unlike other forms of play—the developmental trajectory of construction does not follow a traditional inverted-U developmental function^14^. Rubin and colleagues considered construction as play to involve the manipulation of objects to create something, which has been used to generate massive amounts of descriptive data on how young children use objects (for a full review, see Rubin et al.)^110^.

Children demonstrated a broad range of STEM behaviours during play—most frequently exploring mathematical concepts. This finding is consistent with prior research showing that, even in early childhood, children exhibit emerging competencies in number sense, spatial reasoning, and pattern recognition^111,112^. During play, children often engage in classification, numeration, and magnitude comparison^113^, suggesting that mathematical reasoning is spontaneously activated when children interact with open-ended materials. While earlier studies have emphasized verbal expressions of mathematical thinking, our findings demonstrate that non-verbal behaviours also constitute meaningful forms of mathematical engagement^50,114^.

We coded both verbal and non-verbal indicators of children’s mathematical engagement during unstructured play. Zippert et al.^50^ used a coding framework that identified five primary categories of math exploration: enumeration, magnitude, classification, spatial reasoning, and pattern or shape recognition. In their coding, construction is considered under the spatial category^50^. Although their work examined peer-based math exploration and acknowledged construction as relevant behaviour, it did not include problem-solving as a discrete category. In contrast, our coding scheme differentiated between constructing structures, solving problems, and exploring mathematical concepts. These findings highlight the role of unstructured play as a context for applying and extending mathematical knowledge.

### Factors related to children’s STEM engagement

In the loose parts condition, children’s overall cognitive functioning (FSIQ) predicted their STEM Engagement Score. In contrast, in the control condition, only the VCI—which assessed verbal reasoning and language comprehension—predicted variance in STEM Engagement Score. In the loose parts condition, the frequency of children’s STEM behaviours was not significantly associated with any individual cognitive composite scores (VCI, VSI, FRI, WMI or PSI). However, the FSIQ, which integrates these domains into a comprehensive measure of cognitive functioning, demonstrated stronger predictive power.

We were able to observe how different aspects of cognitive functioning were engaged depending on the play materials available. This may suggest that playing with loose parts may draw more broadly on children’s overall cognitive resources, including working memory and fluid reasoning, whereas play with structured, limited-purpose toys like percussion instruments may rely more specifically on children’s verbal abilities in communicating their play ideas. VCI assesses children’s capacity to understand and use language, which may be particularly relevant in the toy percussion instrument condition, where children were most often engaged in explaining their actions and asking questions. These verbal behaviours likely required the use of expressive language, comprehension, and verbal reasoning. In contrast, loose parts afforded more diverse and cognitively demanding opportunities for construction, symbolic transformation, and problem-solving. The selective involvement of VCI in the toy percussion instrument condition suggests that specific cognitive skills may be differentially activated depending on the affordances of the materials.

Children’s EF was not a predictor of their STEM Engagement Score, but did predict their constructing behaviours. This finding was expected, given previous work^59^; moreover, constructing typically requires planning, spatial reasoning, and problem-solving—all tasks that engage EF. In a hierarchical model outlining a child’s EF development in decision-making and planning^51,115^, EF has distinct phases: problem representation, planning, execution, and evaluation. Development of EF skills may be crucial in children’s ability to entertain multiple conflicting mental representations and plan how to proceed, execute, evaluate, and revise their plans—all displayed prominently in play^59,63^. This model provides conceptual and theoretical support for the notion that children’s behaviours with toys with many affordances align with EF’s key aspects. Children not only have to symbolically transform what they see within their everyday material collection but also must remember the roles they assigned and what to do next; hence, working memory is expected to be a predictor^105^. However, we found that FSIQ was a better predictor of children’s STEM engagement with loose parts than working memory alone. Because FSIQ aggregates across multiple domains—verbal, visual-spatial, fluid reasoning, working memory, and processing speed—capturing general cognitive capacity, it may best reflect the broad demands of engaging in STEM behaviours with a set of loose parts. STEM behaviours, especially in the loose parts condition, involved construction, problem-solving, symbolic use, and exploratory reasoning, all of which may be strongly supported by a composite of abilities rather than by isolated executive functioning processes.

Although we coded both verbal and non-verbal STEM behaviours, constructing structures could be non-verbal as opposed to our other STEM behaviour categories. In our study, even when we excluded constructing behaviours from the overall STEM Engagement Score, children were still involved in significantly more STEM behaviours with loose parts compared to the control. To examine whether construction behaviours disproportionately influenced the overall STEM Engagement Score, we explored the overall STEM engagement with construction removed from the composite score we created. The results demonstrated that the pattern of predictors remained consistent across both conditions. In the control condition with toy percussion instruments, verbal comprehension continued to significantly predict children’s STEM engagement, whether construction was included in the outcome measure or not. Similarly, in the loose parts condition, cognitive functioning (FSIQ) remained a significant predictor in both models, even after removing construction from the composite. Although effect sizes and explained variance were modestly reduced in the adjusted models, the overall conclusions did not change. These findings suggest that, rather than engagement in construction alone, children’s general cognitive abilities accounted for individual differences in STEM engagement. Other researchers have included construction either under the mathematical exploration or as an engineering behaviour in their studies^12,50^. Thus, a STEM coding schema with constructing behaviour provided a comprehensive set of STEM behaviours that may occur in play.

Children’s construction behaviours during play were positively associated with both EF and parental play attitudes and engagement (Factor 4). Play is a culturally mediated activity that varies significantly across sociocultural contexts, shaped by parents’ beliefs, values, and child-rearing practices^116^. This factor included parents’ beliefs about the importance of play, their enjoyment of design and building activities, and the frequency with which they engaged in board or card games and pretend play with their children.

These findings suggest that constructing behaviours are more likely to emerge when children possess the cognitive skills to plan and organize their actions^51^, and when they experience a positive attitude and engagement in their home environment that supports diverse play experiences^79^. Mannweiler and colleagues found that parents’ play strategies are associated with preschoolers’ STEM skill development^117^. Specifically, when parents model STEM-related language and conceptual framing and prioritize play as a meaningful context for learning, children are more inclined to explore, manipulate, and construct with available materials^116^.

Together, these findings highlight the interplay between internal (i.e., EF) and contextual (i.e., family play culture) factors in supporting children’s construction behaviours. They also point to the importance of home learning environments that actively scaffold early STEM engagement. Notably, we did not find significant associations between construction behaviours and other indicators of the home learning environment or parental education. This may be attributable to the negatively skewed distribution of parental education in our sample, as discussed in the limitations.

## Limitations

Several limitations warrant consideration when interpreting the findings of this study. First, while the within-subjects experimental design strengthens internal validity by controlling individual differences, the play sessions were time-limited and conducted in a structured environment, which may not fully capture the complexity or spontaneity of children’s naturalistic play at home or in early learning settings^118^. Future research should extend these findings by conducting longitudinal or observational studies within early learning and childcare settings or homes to assess how STEM behaviours with loose parts are across contexts. Second, although our observational coding captured a broad range of STEM behaviours, the study primarily focused on overt actions and verbalizations. Additional cognitive processes related to problem-solving or planning undoubtedly occurred internally and were not observable. Incorporating complementary methodologies such as think-aloud protocols or child interviews could offer a richer account of children’s reasoning during play.

Additionally, despite efforts to recruit a diverse sample, the participants’ parental education and household income levels were relatively high. Our sample reflected a predominantly urban Canadian context. Although this offers insight into a particular educational and cultural setting, it limits the generalizability of the findings. Future studies should explore children’s STEM behaviours with loose parts across more diverse populations, including families from varied cultural, linguistic, and economic backgrounds. Such research is critical for understanding how social and cultural capital intersect with material affordances to shape STEM engagement in early childhood.

Third, while cognitive functioning was identified as a significant predictor, other influential variables, such as children’s prior familiarity with materials or cultural perceptions of STEM, were not examined. These contextual dimensions may moderate children’s engagement and should be systematically explored in future research to inform more inclusive and culturally responsive approaches. Finally, although loose parts were broadly categorized as versatile, there may be meaningful variation in their material properties (e.g., texture, size, familiarity) that differentially afford STEM behaviours. Particularly, in our methods, the technology dimension was not rich, which may have prevented children from including ideas related to technology in their explorations and thinking. Future research could systematically examine how different combinations of loose parts with different material characteristics and other toy sets with a STEM focus could influence children’s STEM behaviours and learning outcomes. These limitations suggest the need for a more nuanced, context-sensitive understanding of how children interact with everyday objects to explore STEM ideas and innovations. Future studies should aim to bridge controlled experimental approaches with ecologically valid designs and include more diverse samples to strengthen generalizability that can inform and support equitable pedagogical practices.

## Conclusions

Despite growing advocacy for loose parts in early childhood settings, studies into their specific contributions to children’s STEM learning remain limited^2^. More critically, there have been few observational and quantitative studies involving loose parts^4^. Much of the current literature rests on theoretical claims or qualitative studies, leaving a significant gap in understanding how loose parts shape STEM engagement in young children through systematic observations in play. Notably, few studies have examined the extent to which specific STEM behaviours, such as constructing, goal setting, or explaining causal mechanisms, emerge more frequently in the context of open-ended play compared to play with more constrained, single-purpose toys. Moreover, assumptions that all children benefit equally from loose parts overlook potential variability driven by individual cognitive differences or family-level contextual factors. This lack of precision obscures both how and for whom loose parts play a role in facilitating STEM learning, exploration, and innovation.

To address these gaps, the present study employed a within-subjects experimental design to examine children’s STEM behaviours in two contrasting play contexts: one using loose parts and the other using limited-function percussion instruments. Drawing on systematic behavioural observations, we assessed the frequency and nature of children’s STEM behaviours, while also incorporating standardized cognitive and EF measures and parent-reported home learning environments. This integrated approach enables a more differentiated understanding of the conditions under which open-ended materials promote STEM engagement, and which child-level characteristics moderate these effects.

This study has three key contributions. First, through Wilcoxon signed-rank tests, it provides empirical evidence that loose parts elicit specific STEM behaviours not typically observed in play with limited-purpose toys. Second, it reveals that children’s cognitive functioning and, to a lesser extent, parental attitudes, predict the extent of their STEM engagement, raising important questions about equity and access in play-based learning environments. Finally, by challenging the presumption of universal benefit, this study underscores the need for more targeted and developmentally informed approaches to integrating open-ended materials like loose parts into early childhood STEM education. These findings carry direct implications for curriculum development and educator training aimed at fostering equitable and effective STEM learning from the earliest years.

## Supplementary information

Supplementary Information

Supplementary Information

Supplementary Information

## Data Availability

The data supporting the findings of this study are not publicly available to protect participant confidentiality and comply with the conditions of ethical approval. The ethical clearance granted by the MacEwan University Research Ethics Board does not permit open-access data sharing. However, anonymized data have been included on the Open Science Framework (OSF; https://osf.io/tm2s6) solely for data analysis replication purposes.

## Code availability

All analyses were conducted in JASP (version 0.19.3.0). The coding framework and the data used for data analysis are available on the Open Science Framework (OSF) for the purpose of data analysis replication: https://osf.io/tm2s6.
