# Supplementary material for: Loose parts play encourages spontaneous science, technology, engineering, and mathematics (STEM) behaviours
Source: Commun Psychol. 2025 Dec 5;3:183. doi: 10.1038/s44271-025-00362-y (PMC12695631; doi:10.1038/s44271-025-00362-y)
Supplement: Supplementary file 2 — Supplementary Information [file 44271_2025_362_MOESM2_ESM.pdf]

Supplementary Information

Loose Parts Play Encourages Spontaneous Science, Technology, Engineering, and Mathematics (STEM) Behaviours

Dr. Ozlem Cankaya, cankayao@macewan.ca
Department of Early Learning and Curriculum Studies, MacEwan University
Edmonton, AB, Canada

Dr. Natalia Rohatyn-Martin, rohatynmartinn@macewan.ca
Department of Health and Human Services, MacEwan University
Edmonton, AB, Canada

Prof. Karen Buro, burok@macewan.ca
Department of Mathematics and Statistics, MacEwan University
Edmonton, AB, Canada

Prof. Okan Bulut, bulut@ualberta.ca
Department of Educational Psychology, University of Alberta
Edmonton, AB, Canada

Keirsten Taylor, keirsten.taylor@ucalgary.ca
Department of Educational Psychology, University of Calgary
Calgary, AB, Canada

Corresponding Author:
Dr. Ozlem Cankaya, cankayao@macewan.ca
10700 104 Ave NW, Edmonton, AB T5H 0K9 Canada

Supplementary Note 1

Exploratory Factor Analysis and Composite Score Development

Exploratory factor analysis was conducted in JASP to identify underlying patterns among variables by focusing on the shared variance that reflects psychological constructs (See Supplementary Table 2). The analysis identified six factors explaining 46.7% of the variance in the parental questionnaire data. The composite scores for these factors were computed and used for further analysis.

Supplementary Table 1

Summary of Exploratory Factor Analysis Results for Home Learning Environment Variables

Factor	Description	Variance Explained (%)
Frequency of Home Learning Activities (Factor 1)	Included parents' reports on 12 learning activities, such as pointing while reading, introducing words, and teaching letter sounds.	14.5
Parental STEM Attitudes (Factor 2)	Included parents' perceptions of their own math and science abilities, confidence in using technology, and their enjoyment or avoidance of math.	8.5
Frequency of Home Numeracy Activities (Factor 3)	Included engagement in number games, simple sums, and mental math.	7.9
Parental Play Attitudes and Engagement (Factor 4)	Included parents' perspectives on play, enjoyment of building activities, and engagement in board games and pretend play.	5.7
Home Literacy Environment (Factor 5)	Included the number of books, bedtime reading routines, and unstructured play opportunities.	5.2
Parental Literacy Attitudes (Factor 6)	Included enjoyment of reading and writing, confidence in language skills, and a negative association with screen time.	5.0

Predictors and potential covariates of STEM behaviours were examined using linear regressions. These covariates included the child's age in months, sex, parental education

(Anders et al., 2012), cognitive skills measured by various composite scores (i.e., WPPSI-IV's VCI, VSI, FRI, WMI, PSI, and FSIQ), and EF performance (i.e., HTKS Task).

Our within-subjects design allowed each child to experience both conditions; the order of the play conditions was randomly assigned. The play session order was also tested as a covariate to determine whether the sequence in which children were exposed to materials affected their STEM behaviours due to effects such as fatigue, increased familiarity, or a preference for the materials presented earlier. This approach ensured that observed differences in STEM behaviours can be attributed to the materials or activities rather than other factors.

Supplementary Note 2

Factors Predicting STEM Engagement (Excluding Constructing Structures)

An additional set of linear forward selection regressions was conducted to examine predictors of children's STEM Engagement Score when we excluded constructing structures. This analysis aimed to determine whether the other 10 STEM behaviours, which may rely more on children's verbal capacities, in contrast to mostly action-based and non-verbal construction behaviours, would be predicted by a different set of factors.

A linear regression analysis was conducted to examine predictors of children's STEM Engagement Score (excluding constructing structures) while using toy percussion instruments (control). The final model accounted for 8.1% of the variance (R^2 = .08, adjusted R^2 = .06, RMSE = 0.373) and was statistically significant, $F(1, 50) = 4.42$, $p = .040$. VCI predicted STEM Engagement, $B = 0.007$, $SE = 0.003$, $\beta = 0.29$, $t(50) = 2.10$, $p = .040$. The intercept was not statistically significant ($B = -0.36$, $SE = 0.35$, $t(50) = -1.03$, $p = .308$), indicating that variation in STEM Engagement Score was largely attributed to the predictor. Covariates tested but excluded from the final model were child age, sex, EF performance, other cognitive functioning composite

scores (i.e., VSI, FRI, WMI, PSI, FSIQ), play session order, and home learning environment factors. In the loose parts condition, the final model explained 7.7% of the variance (R^2 = .08, adjusted R^2 = .06, RMSE = 0.202) and was statistically significant, F(1, 50) = 4.15, p = .047. Cognitive functioning (FSIQ) emerged as a significant predictor, B = 0.002, SE = 0.001, β = 0.28, t(50) = 2.04, p = .047, indicating that higher overall cognitive ability (FSIQ) is positively associated with higher STEM Engagement Score (excluding construction) with loose parts. The intercept was not statistically significant (B = 0.28, SE = 0.36, t(50) = 0.78, p = .437). Covariates tested but excluded from the final model were the child's age, sex, EF performance, parental education, other cognitive functioning composite scores (i.e., VCI, VSI, FRI, WMI, PSI), play session order, and home learning environment factors. See Supplementary Figure 1 for the relationship between significant predictors and STEM Engagement Scores.

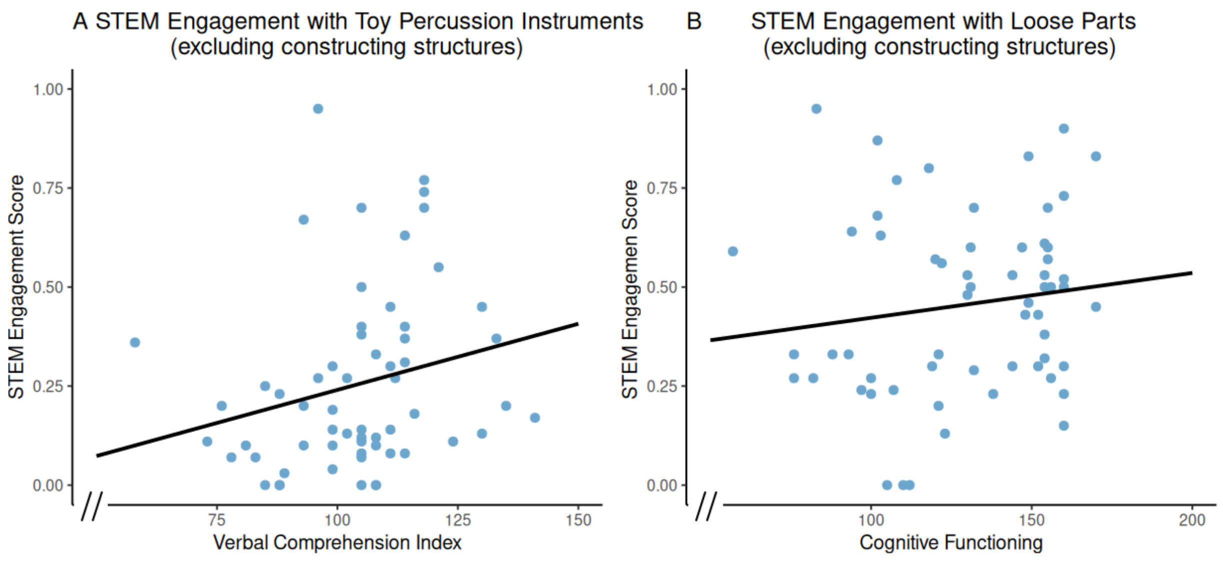

Supplementary Figure 1. Relationships Between Significant Predictors of STEM Engagement Scores (excluding constructing structures). Panel A indicates the relationship between children's STEM Engagement Scores (excluding constructing structures) in the control condition and the predictor VCI. Panel B indicates the relationship between children's STEM

Engagement Score (excluding constructing structures) in the loose parts condition and the predictor cognitive functioning. $N = 52$
