# Supplementary material for: Loose parts play encourages spontaneous science, technology, engineering, and mathematics (STEM) behaviours
Source: Commun Psychol. 2025 Dec 5;3:183. doi: 10.1038/s44271-025-00362-y (PMC12695631; doi:10.1038/s44271-025-00362-y)
Supplement: Supplementary file 1 — Supplementary Information [file 44271_2025_362_MOESM1_ESM.pdf]

nature portfolio

Peer Review File

Loose Parts Play Encourages Spontaneous Science, Technology, Engineering, and Mathematics (STEM) Behaviours

Corresponding Author: Dr Ozlem Cankaya

This file contains all editorial decision letters in order by version, followed by all author rebuttals in order by version.

Version 0:

Decision Letter:

** Please ensure you delete the link to your author homepage in this e-mail if you wish to forward it to your coauthors **

Dear Dr Cankaya,

Thank you for your patience during the peer-review process. Your manuscript titled "Children's Spontaneous Science, Technology, Engineering, and Mathematics (STEM) Behaviours and Engagement in Play with Loose Parts" has now been seen by 2 reviewers, and I include their comments at the end of this message. They find your work of interest but raised some important points. We are interested in the possibility of publishing your study in Communications Psychology, but would like to consider your responses to these concerns and assess a revised manuscript before we make a final decision on publication.

We therefore invite you to revise and resubmit your manuscript, along with a point-by-point response to the reviewers. Please highlight all changes in the manuscript text file.

To strengthen the manuscript, the following key issues must be addressed in revision:

1. Given the large number of exploratory comparisons and the absence of preregistration, please clearly separate the Bonferroni-corrected t-test findings from the broader exploratory analyses, and interpret them accordingly. These distinctions should be made explicit in the Results, Discussion, and Conclusions.

2. Please include graphical depictions (e.g., scatterplots or boxplots) of the main findings to allow readers to assess variability, effect size, and distribution. This will greatly enhance transparency and interpretability.

3. Both reviewers noted that the observed STEM behaviors related to construction may be driven by the specific affordances of the blocks used on construction. Please clarify how much of the effect of loose parts play on STEM behaviors is attributable to blocks specifically. This should be addressed both analytically (as per reviewer suggestions) and conceptually, with attention to the STEM-conducive affordances of the objects used.

4. The introduction is currently 13 pages long and structured like a review. Please shorten it to focus more directly on the research question and rationale for the current study.

5. Please ensure you follow our statistical guidelines when reporting statistics (https://www.nature.com/commspsychol/submit/submission-guidelines#statistical-guidelines). Please note in particular our requirements for the reporting and interpretation of null-results. Please use relevant methods, such as Bayesian statistics or equivalence tests, to support the reported null result regarding gender, which is an interesting and potentially meaningful finding.

We are committed to providing a fair and constructive peer-review process. Please don't hesitate to contact us if you wish to discuss the revision in more detail.

I am attaching an Editorial Requests Table that details critical reporting requirements for the revised manuscript. Please attend to each item and ensure your manuscript is fully compliant. If your revised manuscript is not aligned with these requests on major issues, such as those concerning statistics, it may be returned to you for further revisions without re-review.

Please submit the following items:

- Revised manuscript
- Point-by-point response to the referees' comments
- Cover letter (as a separate document)
- Nature Research Reporting Summary
- Completed Editorial Request Table (attached).

via this link: Link Redacted .

** This url links to your confidential home page and associated information about manuscripts you may have submitted or be reviewing for us. If you wish to forward this email to co-authors, please delete the link to your homepage first **

Additional guidance is available in our style and formatting guide Communications Psychology formatting guide.

We hope to receive your revised paper within 8 weeks; please let us know if you aren't able to submit it within this time so that we can discuss how best to proceed. If we don't hear from you, and the revision process takes significantly longer, we may close your file. In this event, we will still be happy to reconsider your paper at a later date, provided it still presents a significant contribution to the literature at that stage.

We would appreciate it if you could keep us informed about an estimated timescale for resubmission, to facilitate our planning.

We look forward to seeing the revised manuscript and thank you for the opportunity to review your work.

Best regards,

Shir Atzil

Shir Atzil
External Editor
Communications Psychology

REVIEWER EXPERTISE:
Reviewer #1: Early childhood education, play
Reviewer #2: Play and early education

REVIEWER REPORTS:

Reviewer #1 (Remarks to the Author):

Thank you for providing this manuscript that investigates the role of "loose parts" in children's play and its relation to early STEM learning.

This is an important piece, as it demonstrates the potential of play with everyday objects and low-tech toys, which can still promote STEM without the need for expensive gadgets. The paper has a broad appeal for developmental psychology, STEM education, early childhood education, and parenting.

INTRODUCTION

The focus on "loose parts" also provides a bridge between debates in the field of play and learning and discussions on the role of object play vs. imaginative play forms. As children play with loose parts, they interject conceptual understanding into objects. For a side of this debate, see:

Lillard, A.S. and Taggart, J. (2019), Pretend Play and Fantasy: What if Montessori Was Right?. Child Dev Perspect, 13: 85-90. https://doi-org.ep.bib.mdh.se/10.1111/cdep.12314

The authors may want to consider this notion.

The authors aim to study the "capacities and scaffold meaningful STEM engagement prior to the introduction of formal instruction or exposure to advanced STEM domains (e.g., robotics, coding)." This is an important notion for children as an entryway into STEM, but also note that the type of free play behaviour studied here can be an entry also into these "advanced STEM domains", see:

Samuelsson, R. (2024). Learning beyond instructionist/constructionist divides: A mixed methods exploration of three learning designs for 1-2-year-old children. Computers & Education, 218, 105089. https://doi.org/10.1016/j.compedu.2024.105089

METHODS
Overall, the study design is excellent and well described. However, the authors should provide a further rationale for the choice of Box A items in the design. These items are unrelated to STEM. Thus, it would be expected that Box B, with items such as building blocks, would be more conducive to STEM, as shown in the results for "constructing structures". For example, including a STEM toy for one group would have made the results more surprising. The STEM-conducive affordances of these objects should be discussed more.

Reviewer #2 (Remarks to the Author):

• Literature review – I find that it’s more meaning as a reader to know the nature or type of studies that you are reviewing instead of whether the study adopts quantitative or qualitative methodology. Both methodologies are valuable in producing evidence and contribute to knowledge. In this case, I suggest for the authors to remove statements such as “strong theoretical and qualitative enthusiasm” on page 4; “Explicit quantitative research” on page 5, “Foundational research” on page 5. More precise type of findings should be zoomed into to direct readers’ attention to the gaps of these studies.

• Literature review - Ensure citations provided are necessary and align with statements. Eliminate redundant citations from your review, such as Beloglovsky & Daly, 2016 on page 4 for a statement that define loose parts.

• Literature review – Ensure accuracy of authors’ names and year of publication in citations, for example, Zheng and Ng (2024) on page 6 should corrected to Zeng and Ng (2024).

• Literature review – On pages 6 to 7, the authors had commented repeatedly on generalizability; I believe that this should not be emphasized on as every published research study / article has its own merits and contribute to the wider knowledge base in one way or another. A better way is to focus on the knowledge gap instead of methodological design.

• Literature review – I appreciate that each section is very informative and generally clearly written. Taking into account of the word count and to sustain readers’ attention, do reduce the review and make the contents more concise.

• Methodology – The data collection and analysis methods were well-planned and rationalised. I find the proposed STEM Play Behaviour Scale interesting and would contribute valuable knowledge to early childhood educational settings. I am just wondering about the category on “Using STEM-Specific Language” as vocabulary words relating to STEM can be very broad, and majority of words verbalised during STEM activities could be coded for this category . What are some words that do not constitute this category of behaviour?

• Findings – In Table 5, the segregation of Construction in STEM engagement score was confusing to me as the previous tables had analysed in terms of two groups – loose parts and control. The same should apply for Table 5. From an educator perspective, STEM does not consist of solely building or construction activities. I acknowledge that the children you had observed engaged in constructing activities significantly more than other activities. You could make these more interesting by excluding different types of activities, such as maths-related ones, instead of solely construction in order to explore the differences in STEM engagement activities.

• Findings – Following up on the earlier point on constructing activities, Constructing Structures is considered as a STEM behaviour in terms of an action. However, the other STEM behaviour mostly constitute soft/cognitive skills or verbalisation skills, such as solving problems and asking questions. I am keen to see the findings for when constructing structures is removed from your scale to consist of only the soft/cognitive skills and types of verbalisation, how will this affect the overall results?

• Findings – In Table 6, what was the reason for including home literacy environment and parental literacy attitudes in the parents’ questionnaire? There was no explanation or review on the link to STEM and loose parts play in the literature review

section. The connection amongst these aspects could be made clearer in the literature review sections. If these two factors were removed from the factor analysis, how will this influence the factors' impact on children's outcomes and STEM behaviours?

• Discussion – Discussion is clearly written but the results might change if the authors explore the two suggestions on the removal of Constructing Structures in the scale and the two factors in the factor analysis.

* TRANSPARENT PEER REVIEW: Communications Psychology uses a transparent peer review system. This means that we publish the editorial decision letters including Reviewers' comments to the authors and the author rebuttal letters online as a supplementary peer review file. However, on author request, confidential information and data can be removed from the published reviewer reports and rebuttal letters prior to publication. If your manuscript has been previously reviewed at another journal, those Reviewers' comments would not form part of the published peer review file.

** Visit Nature Research's author and referees' website at www.nature.com/authors for information about policies, services and author benefits**

Communications Psychology is committed to improving transparency in authorship. As part of our efforts in this direction, we are now requesting that all authors identified as 'corresponding author' create and link their Open Researcher and Contributor Identifier (ORCID) with their account on the Manuscript Tracking System prior to acceptance. ORCID helps the scientific community achieve unambiguous attribution of all scholarly contributions. You can create and link your ORCID from the home page of the Manuscript Tracking System by clicking on 'Modify my Springer Nature account' and following the instructions in the link below. Please also inform all co-authors that they can add their ORCIDs to their accounts and that they must do so prior to acceptance.
https://www.springernature.com/gp/researchers/orcid/orcid-for-nature-research

For more information please visit http://www.springernature.com/orcid

If you experience problems in linking your ORCID, please contact the Platform Support Helpdesk.

Version 1:

Decision Letter:

** Please ensure you delete the link to your author homepage in this e-mail if you wish to forward it to your coauthors **

Dear Dr Cankaya,

Your manuscript titled "Children's Spontaneous Science, Technology, Engineering, and Mathematics (STEM) Behaviours and Engagement in Play with Loose Parts" has now been seen by our reviewers, whose comments appear below. In light of their advice I am delighted to say that we are happy, in principle, to publish a suitably revised version in Communications Psychology.

We therefore invite you to revise your paper one last time to address the remaining concerns of our reviewers and a list of editorial requests. At the same time we ask that you edit your manuscript to comply with our format requirements and to maximise the accessibility and therefore the impact of your work.

EDITORIAL REQUESTS:

Please review our specific editorial comments and requests regarding your manuscript in the attached "Editorial Requests Table". Please outline your response to each request in the right hand column. Please upload the completed table with your manuscript files as a Related Manuscript file.

If you have any questions or concerns about any of our requests, please do not hesitate to contact me.

SUBMISSION INFORMATION:

In order to accept your paper, we require the files listed here https://www.nature.com/documents/commsj-file-checklist.pdf .

OPEN ACCESS:

Communications Psychology is a fully open access journal. Articles are made freely accessible on publication. For further information about article processing charges, open access funding, and advice and support from Nature Research, please visit https://www.nature.com/commspsychol/open-access

At acceptance, you will be provided with instructions for completing the open access licence agreement on behalf of all authors. This grants us the necessary permissions to publish your paper. Additionally, you will be asked to declare that all required third party permissions have been obtained, and to provide billing information in order to pay the article-processing charge (APC).

* TRANSPARENT PEER REVIEW: Communications Psychology uses a transparent peer review system. On author request, confidential information and data can be removed from the published reviewer reports and rebuttal letters prior to publication. If you are concerned about the release of confidential data, please let us know specifically what information you would like to have removed. Please note that we cannot incorporate redactions for any other reasons.

* CODE AVAILABILITY: All Communications Psychology manuscripts must include a section titled "Code Availability" at the end of the methods section. We require that the custom analysis code supporting your conclusions is made available in a publicly accessible repository at this stage; please choose a repository that generates a digital object identifier (DOI) for the code; the link to the repository and the DOI must be included in the Code Availability statement. Publication as Supplementary Information will not suffice.

* DATA AVAILABILITY:
All Communications Psychology manuscripts must include a section titled "Data Availability" at the end of the Methods section. More information on this policy, is available in the Editorial Requests Table and at http://www.nature.com/authors/policies/data/data-availability-statements-data-citations.pdf.

Please use the following link to submit the above items:
Link Redacted
** This url links to your confidential home page and associated information about manuscripts you may have submitted or be reviewing for us. If you wish to forward this email to co-authors, please delete the link to your homepage first **

We hope to hear from you within two weeks; please let us know if you need more time.

Best regards,

Jennifer Bellingtier

Jennifer Bellingtier, PhD
Senior Editor
Communications Psychology

Shir Atzil
External Editor
Communications Psychology

REVIEWERS' EXPERTISE:

Reviewer #1: Early childhood education, play
Reviewer #2: Play and early education

REVIEWERS' COMMENTS:

Reviewer #1 (Remarks to the Author):

Thank you for providing an updated manuscript with clear responses to the reviewer comments. The authors have taken great care in revising the manuscript, making it a more coherent and well-rounded addition to the literature. I look forward to seeing it published.

Reviewer #2 (Remarks to the Author):

I am generally satisfied with the revision, and the only comment I have would be to explain Figure 2 more in your Findings section. In addition, you would want to consider reducing the length of your findings as well as discussion to make it more concise.

** Visit Nature Research's author and referees' website at www.nature.com/authors for information about policies, services and author benefits**

Open Access This Peer Review File is licensed under a Creative Commons Attribution 4.0 International License, which permits use, sharing, adaptation, distribution and reproduction in any medium or format, as long as you give appropriate credit to the original author(s) and the source, provide a link to the Creative Commons license, and indicate if changes were made.
In cases where reviewers are anonymous, credit should be given to 'Anonymous Referee' and the source.
The images or other third party material in this Peer Review File are included in the article’s Creative Commons license, unless indicated otherwise in a credit line to the material. If material is not included in the article’s Creative Commons license and your intended use is not permitted by statutory regulation or exceeds the permitted use, you will need to obtain permission directly from the copyright holder.
To view a copy of this license, visit https://creativecommons.org/licenses/by/4.0/

Nature Communications Psychology
Manuscript COMMSPSYCHOL-25-0523

Editor Comments and Suggestions	Author Responses and Explanations	Revisions Implemented
Thank you for your patience during the peer-review process. Your manuscript titled "Children's Spontaneous Science, Technology, Engineering, and Mathematics (STEM) Behaviours and Engagement in Play with Loose Parts" has now been seen by 2 reviewers, and I include their comments at the end of this message. They find your work of interest but raised some important points.<br><br>We are interested in the possibility of publishing your study in Communications Psychology, but would like to consider your responses to these concerns and assess a revised manuscript before we make a final decision on publication. We therefore invite you to revise and resubmit your manuscript, along with a point-by-point response to the reviewers. Please highlight all changes in the manuscript text file. To strengthen the manuscript, the following key issues must be addressed in revision:	We appreciate the opportunity to revise and resubmit and believe the manuscript has been significantly strengthened by the constructive feedback you provided.	We inserted page numbers in the manuscript to enable precise cross-referencing between reviewer comments and our revisions. Although the manuscript includes MS Word track changes, we also highlighted newly added statements and/or sections to facilitate easy identification whenever possible.
1. Given the large number of exploratory comparisons and the absence of	N/A	In response, we revised the Results (page 23), Discussion (page 37), and Conclusion

preregistration, please clearly separate the Bonferroni-corrected t-test findings from the broader exploratory analyses, and interpret them accordingly. These distinctions should be made explicit in the Results, Discussion, and Conclusions.		(page 48) sections by either introducing explicit subtitles or indicating the Wilcoxon signed-rank tests to clearly distinguish the t-test findings from other exploratory analyses. We also revised the interpretation of each set of results accordingly. In the revised manuscript, these changes are highlighted in yellow.
2. Please include graphical depictions (e.g., scatterplots or boxplots) of the main findings to allow readers to assess variability, effect size, and distribution. This will greatly enhance transparency and interpretability.	N/A	We have added a figure with boxplots on page 28 to illustrate group differences from the Bonferroni-corrected Wilcoxon signed-rank test analysis.<br>**Figure 2. Boxplots comparing children's play behaviours, engagement, and play session duration with toy percussion instruments (control) and loose parts.** Panels A–F display children's STEM behaviours, STEM Engagement Score, and play session duration for the control and loose parts sessions. Only variables significantly different between conditions are included.<br><br>For the regression analyses, we added scatterplots to depict the relationships between predictor and outcome variables. Here are the added figures and page numbers:<br>On page 30, **Figure 3. Mean Proportions of Time for STEM Behaviours.** Panel A displays the proportions of each STEM

		behaviour in the control condition. Panel B displays proportions of each STEM behaviour in the loose parts condition. In panels A and B, the mean proportion of each STEM behaviour is marked with a red diamond.<br><br>On page 33, **Figure 4. Relationships Between Significant Predictors of STEM Engagement Scores.** Panel A indicates the relationship between children's STEM Engagement Scores in the control condition and the predictor VCI. Panel B indicates the relationship between children's STEM Engagement Score in the loose parts condition and the predictor cognitive functioning.<br><br>On page 35, **Figure 5. Relationships Between Significant Predictors of STEM Engagement Scores (excluding constructing structures).** Panel A indicates the relationship between children's STEM Engagement Scores (excluding constructing structures) in the control condition and the predictor VCI. Panel B indicates the relationship between children's STEM Engagement Score (excluding constructing structures) in the loose parts condition and the predictor cognitive functioning.<br>On Page 36, **Figure 6. Relationships Between Significant Predictors of Constructing Structures with Loose**

		**Parts.** Panel A displays the relationship between children's STEM behaviour of constructing structures in the loose parts condition and the predictor EF performance. Panel B displays the relationship between children's STEM behaviour of constructing structures in the loose parts condition and the predictor parental play attitudes.
3. Both reviewers noted that the observed STEM behaviours related to construction may be driven by the specific affordances of the blocks used in construction. Please clarify how much of the effect of loose parts play on STEM behaviors is attributable to blocks specifically. This should be addressed both analytically (as per reviewer suggestions) and conceptually, with attention to the STEM-conducive affordances of the objects used.	Thank you for raising this important point and giving us an opportunity to explain our reasoning conceptually. First, we would like to note that the wooden blocks we used were not standard building blocks but unconventional components from a marble run set (see Figure 1 below), selected intentionally to avoid privileging any particular play type (i.e., constructive play). Their varied shapes and holes allowed versatile use. For instance, a long block with a groove (e.g., a channel carved) could be interpreted by children as a bed in pretend play if they perceived that affordance (see Figure 2 below). Second, blocks made up only 22% of the total loose parts we used, which included a wide range of textures and materials (e.g., wood, foam, cork, plastic, glass). The foam cylinder and cubes we included were not ideal for stacking, as they needed intricate balancing	To respond to this comment analytically, we conducted an additional regression analysis section titled "Factors Predicting STEM Engagement (Excluding Constructing Structures)", where we took out construction, in addition to exploring the predictors of children's construction separately. The predictors of overall stem engagement score did not change. Please see further details in our explanation below responding to Reviewer #2, point #8.

	and did not stay on top of each other easily. The materials were selected in consultation with 10 early childhood education faculty members/researchers and educators to ensure they represented the kind of versatile loose parts typically accessible to children, varying in material and texture, with a blend of natural and manufactured materials. Third, many scholars classify blocks as a type of loose part; for example, Gold et al. (2021) examined engineering behaviours in children using only blocks as loose parts. In 2025, we published a systematic review on the relationship between loose parts and children's cognitive development. One of our inclusion criteria for research studies with blocks to be included as loose parts was to be used in conjunction with other versatile materials, not by themselves. This was described in our review(Cankaya et al., 2025) as "For instance, studies that investigated single materials such as blocks, LEGO, Duplo, Magna-Tiles, Lincoln Logs, Imagination Playground blocks, and Playmobil were not included. We only included branded toys, such as LEGO, DUPLO, Play-Doh, Lite-Brite, etc., when combined with other play materials or toys	

	that could be considered loose parts." The reason for establishing the inclusion criteria was that there are many research studies with blocks. However, we were interested in exploring how children respond to a variety when there are multiple materials combined.<br><br>References:<br>Cankaya, O., Martin, M., & Haugen, D. (2025). The Relationship Between Children's Indoor Loose Parts Play and Cognitive Development: A Systematic Review. *Journal of Intelligence*, *13*(5), 52.<br><br>Gold, Z. S., Elicker, J., Evich, C. D., Mishra, A. A., Howe, N., & Weil, A. E. (2021). Engineering play with blocks as an informal learning context for executive function and planning. *Journal of Engineering Education*, *110*(4), 803-818.	

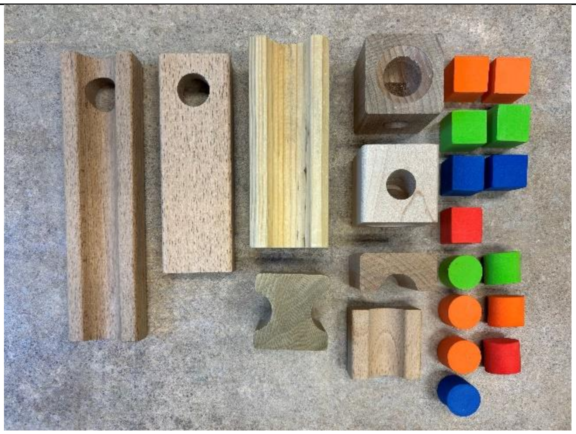

Figure 1. *The foam (colourful) and wooden blocks were 22 percent of all loose parts in Box B, and the wooden blocks had unconventional shapes.*

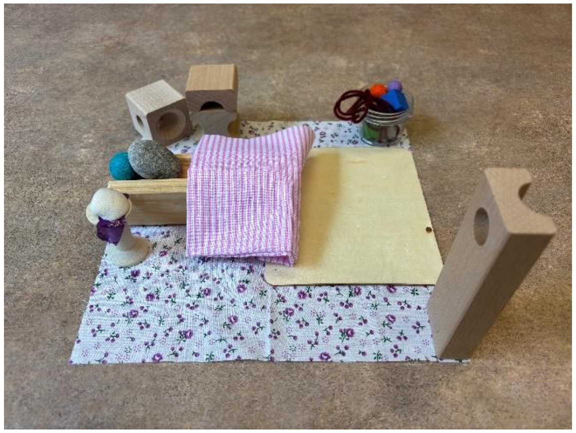

Figure 2. *Potential made up use of blocks as bedroom furniture in a pretend play scenario.*

4. The introduction is currently 13 pages long and structured like a review. Please shorten it to focus more directly on the research question and rationale for the current study.	We worked with a professional editor to focus more directly on the research question and rationale for the current study.	We removed some of the literature review content and streamlined the section to emphasize the context, rationale, the gaps in the literature, and the study's contributions. The revised Introduction is now 6.5 pages long (we reduced the word count from 3975 to 1945, a 51 percent reduction). The parts that are eliminated are marked with MS Word's track changes function (discussed with Drs. Bellingtier)**.** This word count will be further reduced if numbering is used for citations.
5. Please ensure you follow our statistical guidelines when reporting statistics (https://www.nature.com/commspsychol/submit/submission-guidelines#statistical-guidelines). Please note in particular our requirements for the reporting and interpretation of null-results.<br><br>Please use relevant methods, such as Bayesian statistics or equivalence tests, to support the reported null result regarding gender, which is an interesting and potentially meaningful finding.	N/A	We have closely followed the guidelines in our reporting. We are willing to make changes if there are parts that need further work.<br>We created a separate section titled "Examining Sex Differences in Cognitive Development and STEM Engagement" on page 23 and provided details with Bayesian statistics to support the reported null result regarding sex effects. Our statement in the original submission was supported. We also changed the wording boy/girl as they relate to gender; instead, we used male/female following SAGER guidelines: https://doi.org/10.1186/s41073-016-0007-6
I am attaching an Editorial Requests Table that details critical reporting requirements for the revised manuscript. Please attend to	N/A	We addressed all points to comply with the editorial request table; when it was not applicable, we left it blank.

each item and ensure your manuscript is fully compliant. If your revised manuscript is not aligned with these requests on major issues, such as those concerning statistics, it may be returned to you for further revisions without re-review.		
• Please submit the following items: Revised manuscript<br>• Point-by-point response to the referees' comments<br>• Cover letter (as a separate document)<br>• Nature Research Reporting Summary<br>• Completed Editorial Request Table (attached).<br>via this link: https://mts-commspsychol.nature.com/cgi-bin/main.plex?el=A4DU3mR6A6DVT1I5A9ftdJVuRTUea2NQMB6eWv0qe1wZ .	N/A	We submitted all items listed.
Additional guidance is available in our style and formatting guide Communications Psychology formatting guide.	N/A	We went through our manuscript and formatted it according to the guidelines.

**Reviewer #1 Comments and Suggestions**	**Author Responses and Explanations**	**Revisions Implemented**
1. Thank you for providing this manuscript that investigates the role of "loose parts" in children's play and its	Thank you for your encouragement and recognition of the importance of this research. It motivated us to make the	No action was required.

relation to early STEM learning.<br><br>This is an important piece, as it demonstrates the potential of play with everyday objects and low-tech toys, which can still promote STEM without the need for expensive gadgets. The paper has a broad appeal for developmental psychology, STEM education, early childhood education, and parenting.	necessary changes to strengthen and improve the manuscript.	
2. INTRODUCTION<br>The focus on "loose parts" also provides a bridge between debates in the field of play and learning and discussions on the role of object play vs. imaginative play forms. As children play with loose parts, they interject conceptual understanding into objects. For a side of this debate, see:<br><br>Lillard, A.S. and Taggart, J. (2019), Pretend Play and Fantasy: What if Montessori Was Right?. Child Dev Perspect, 13: 85-90. https://doi-org.ep.bib.mdh.se/10.1111/cdep.12314 The authors may want to consider this notion.	We were familiar with Dr. Lillard's earlier work (Lillard, 2008), and we appreciate the suggestion of this nuanced article on children's preferences for real objects and activities in play. While we did not measure children's liking of the loose parts and toys in the present study, in our subsequent study on parent-child play with loose parts, we asked both parents and children about their preferences. This piece of work will be important for informing and strengthening our forthcoming manuscripts.<br>References:<br>Lillard, A. (2008). How important are the Montessori materials. *Montessori Life*, *20*(4), 20-25.	We incorporated this paper into our introduction on page 6.

3. The authors aim to study the "capacities and scaffold meaningful STEM engagement prior to the introduction of formal instruction or exposure to advanced STEM domains (e.g., robotics, coding)." This is an important notion for children as an entryway into STEM, but also note that the type of free play behaviour studied here can be an entry also into these "advanced STEM domains", see:<br><br>Samuelsson, R. (2024). Learning beyond instructionist/constructionist divides: A mixed methods exploration of three learning designs for 1-2-year-old children. Computers & Education, 218, 105089. https://doi.org/10.1016/j.compedu.2024.105089	Thank you for suggesting the idea that advanced STEM domain knowledge and skills enter children's lives as building blocks and supporting this idea with an article that aligns with our view.	We incorporated Samuelsson's (2024) work into our introduction on pages 4 and 5. We also explained with the same citation how early explorations in free play are the building blocks for the advanced system domains, such as robotics and coding on page 5.
4. METHODS<br>Overall, the study design is excellent and well described. However, the authors should provide a further rationale for the choice of Box A items in the design. These items are unrelated to STEM. Thus, it would be expected that Box B, with items such as building blocks, would be more conducive to	We selected toy percussion instruments as the control condition (Box A) deliberately, knowing that children would not engage in extensive play or STEM-related explorations with them. In contrast, we sought to see, compared to our Box A, how much children engage in STEM behaviours with loose parts. The percussion instruments were multi-pieced and varied in	We added two short sections below(they are highlighted in the manuscripts for easy identification):<br>1. In the Methods section on page 15, we included the following: "We selected toy percussion instruments as the control condition because, while they are multi-piece and varied in texture, they offer limited affordances for play."

STEM, as shown in the results for "constructing structures". For example, including a STEM toy for one group would have made the results more surprising. The STEM-conducive affordances of these objects should be discussed more.	texture (fabric, metal, wood, plastic), which helped control for novelty and sensory stimulation without introducing construction or STEM-specific affordances.<br><br>This ensured that differences in engagement could be attributed to material affordance rather than attractiveness or complexity. We avoided including a STEM-related toy as the control to maintain a clear contrast between open-ended and limited-purpose materials. Adding a STEM toy would also have required a third play session, which could have overloaded children and caused fatigue in our within-subjects design. However, we acknowledge the value of comparing loose parts with explicitly STEM-conducive materials. This is the focus of the next phase of our research, currently under review for funding, which will compare different combinations of loose parts and other toy sets following Hashmi et al.'s (2020) methodology and play material selection where they introduced many parallel boxes.<br><br>Reference:<br>Hashmi, S., Vanderwert, R. E., Price, H. A., & Gerson, S. A. (2020). Exploring the	2. In the Limitations section on page 47, we included a sentence that states, "Future research could systematically examine how different combinations of loose parts with different material characteristics and other toy sets with a STEM focus could influence children's STEM behaviours and learning outcomes."

	benefits of doll play through neuroscience. Frontiers in Human Neuroscience, 14, 560176. https://doi.org/10.3389/fnhum.2020.560176	

**Reviewer #2 Comments and Suggestions**	**Author Responses and Explanations**	**Revisions Implemented**
1. Literature review – I find that it's more meaning as a reader to know the nature or type of studies that you are reviewing instead of whether the study adopts quantitative or qualitative methodology. Both methodologies are valuable in producing evidence and contribute to knowledge.<br>In this case, I suggest for the authors to remove statements such as "strong theoretical and qualitative enthusiasm" on page 4; "Explicit quantitative research" on page 5, "Foundational research" on page 5. More precise type of findings should be zoomed into to direct readers' attention to the gaps of these studies.	Thank you for this suggestion. This suggestion also helped with shortening our background and literature review.	We have removed the phrases or wording noted by Reviewer #2 ("strong theoretical and qualitative enthusiasm," "explicit quantitative research," and "foundational research") and revised the manuscript to describe the specific findings of each study while maintaining our focus on the research gap. These wording changes were also implemented throughout the manuscript; however, we particularly highlighted the word changes on page 7.
2. Literature review - Ensure citations provided are necessary and align with statements. Eliminate redundant citations from your review, such as	N/A.	We combed the introduction and literature review to reduce redundant citations, particularly repeated references to Beloglovsky and Daly (2016) and other

Beloglovsky & Daly, 2016 on page 4 for a statement that define loose parts.		repeated references (e.g., Cankaya et al., 2025; Gull et al., 2024)
3. Literature review – Ensure accuracy of authors' names and year of publication in citations, for example, Zheng and Ng (2024) on page 6 should corrected to Zeng and Ng (2024).	Thanks for catching the typo in Zeng and Ng (2024).	We fixed the typo in the Zeng and Ng (2024) citation. Furthermore, we went through our list of citations and references for accuracy.
4. Literature review – On pages 6 to 7, the authors had commented repeatedly on generalizability; I believe that this should not be emphasized on as every published research study / article has its own merits and contribute to the wider knowledge base in one way or another. A better way is to focus on the knowledge gap instead of methodological design.	One of the gaps we noticed in this domain of research is the lack of quantitative studies with loose parts focusing on children indoor play. While articulating the need for our study, we may have overemphasized the methodologies that were prominent in the field.	We redirected our emphasis to the gap we fill as suggested by the reviewer.
5. Literature review – I appreciate that each section is very informative and generally clearly written. Taking into account of the word count and to sustain readers' attention, do reduce the review and make the contents more concise.	N/A	The literature review was previously 3,975 words. We edited the manuscript for length and conciseness; now it is 1945 words with citations. This word count will be further reduced to 1500 if numbering is used for citations.
6. Methodology – The data collection and analysis methods were well-planned and rationalised. I find the proposed STEM Play Behaviour Scale interesting and would contribute valuable	Thank you for your encouragement and for recognizing the contribution of our work to the field.<br><br>We appreciate this request for clarification regarding the category on "Using STEM-	No action was required.

knowledge to early childhood educational settings.<br><br>I am just wondering about the category on "Using STEM-Specific Language" as vocabulary words relating to STEM can be very broad, and majority of words verbalised during STEM activities could be coded for this category. What are some words that do not constitute this category of behaviour?	Specific Language". While we have not yet transcribed these solitary play sessions, our coding defines *Using STEM-Specific Language* as the use of vocabulary directly related to STEM. There is previous research that specifically explored the STEM language use in play by Thibodeau-Nielsen et al. (2025) we took into consideration while coding.<br><br>Our examples of words during play that are general rather than STEM-specific can be social phrases, emotional expressions, imaginative story elements, which were not coded in this category.<br>We compiled some examples of such words that would *not* be coded as STEM-specific language in solitary play from our data:<br>• General descriptors: "Pretty," "Nice," and "I will put the shell here."<br>• Pretend play: "This is the dragon's house," "The princess is flying.", "This is my doll's blanket."<br>• Emotion: "I like this," "That's fun."	
7. Findings – In Table 5, the segregation of Construction in STEM engagement score was confusing to me as the previous tables had analysed in terms of two groups – loose parts and control.	Thank you for pointing out that isolating construction can be confusing in Table 5 and for explaining your rationale.	To avoid confusion, we removed the "STEM Engagement (excluding Construction)" from Table 5 and reported a single STEM Engagement outcome.

The same should apply for Table 5. From an educator perspective, STEM does not consist of solely building or construction activities. I acknowledge that the children you had observed engaged in constructing activities significantly more than other activities. You could make these more interesting by excluding different types of activities, such as maths-related ones, instead of solely construction in order to explore the differences in STEM engagement activities.		We conducted two additional linear regression analyses for STEM Engagement Score (Excluding Constructing Structures) for loose parts and control. Their contributions to the models for each analysis were slightly different, but significant predictors in our final models were unchanged. We consider this to be a valuable inclusion that shows children's STEM behaviours (whether including or excluding construction) are predicted by the same factors. This also assists in addressing editor comment #3 regarding how STEM behaviours may arise from the affordances of materials that support children's construction.
8. Findings – Following up on the earlier point on constructing activities, Constructing Structures is considered as a STEM behaviour in terms of an action. However, the other STEM behaviour mostly constitute soft/cognitive skills or verbalisation skills, such as solving problems and asking questions. I am keen to see the findings for when constructing structures is removed from your scale to consist of only the soft/cognitive skills and types of verbalisation, how will this affect the overall results?	We acknowledge that some of the STEM behaviours depend more on children's verbalization/soft cognitive skills, as suggested in this comment. We briefly discussed this in our discussion in our original submission. In the background, when we initially explored our data, we also explored whether children's vocabulary was related to their STEM engagement and behaviours (it was not).<br><br>As construction, building, and engineering behaviours are a prominent part of existing research regarding STEM in early	**Wilcoxon signed-rank tests comparisons:** Removing the Constructing Structures from our scale or the t-test comparisons would not impact these results, as we compared each category between loose parts and toy percussion instrument conditions separately.<br><br>**Regression analysis:**<br>To respond to our reviewer's suggestion, we conducted two additional linear regression analyses for STEM Engagement Score (Excluding Construction) for loose parts and control on pages 33-35, with Figure 5 (scatter plots) illustrating the

	childhood, we strived to capture all aspects of STEM to enable our study to be compared to previous findings (e.g., Gold & Elicker, 2020). Thibodeau-Nielsen and colleagues (2025) also explored "building" as one of their language categories, although they only explored the verbal component of STEM activities children were involved in. Lastly, even though our materials were simple and did not include technology (i.e., electronics), we still looked at whether children spontaneously explored technology-related ideas. Additionally, our reasoning for including STEM Engagement (without constructing structures) was to emphasize that children go beyond constructing structures/building with loose parts, and they exhibited a variety of STEM behaviours, even when we excluded constructing behaviours.<br><br>References:<br>Gold, Z. S., & Elicker, J. (2020). Engineering peer play: A new perspective on science, technology, engineering, and mathematics (STEM) early childhood education. In *Peer Play and Relationships in Early Childhood: International Research*	relationships between significant predictors and STEM engagement score (excluding constructing structures). Significant predictors were mainly unchanged and their contributions to the models for each analysis were only slightly changed.<br><br>In our results, we explained and framed these findings with the reviewer's suggestion, which brought depth to our reasoning, illustrating that the STEM behaviours children show through verbal and nonverbal behaviours go beyond the action of constructing, particularly with loose parts.<br><br>If the Reviewer #2 believes that we should keep this analysis, we can leave it in the manuscript in this revised version. If they believe that this analysis is not needed, as the predictors have not changed, we can leave the results and discussion parts out.

	Perspectives (pp. 61-75). Cham: Springer International Publishing.<br><br>Thibodeau-Nielsen, R. B., Rueda-Posada, M. F., Dier, S. E., Dooley, A. W., Nadler, D. R., & Coxon, S. V. (2025). Exploring Playful Opportunities for STEM Learning in Early Elementary School. *Early Education and Development*, 1–16.	
9. Findings – In Table 6, what was the reason for including home literacy environment and parental literacy attitudes in the parents' questionnaire? There was no explanation or review on the link to STEM and loose parts play in the literature review section. The connection amongst these aspects could be made clearer in the literature review sections. If these two factors were removed from the factor analysis, how will this influence the factors' impact on children's outcomes and STEM behaviours?	Our research program explores how various home experiences reflect on children's play behaviours and cognitive development. In North America, parents prioritize literacy and play activities over math and other academic learning opportunities. The home literacy environment and particularly parents' attitudes towards learning literacy and numeracy at home strongly relate to children's overall cognitive outcomes, including science and mathematics achievement (Anders et al., 2012; Jang et al., 2024). Researchers observe a similar pattern in Europe with longitudinal studies finding that home learning environment and parents' focus on literacy in the home environment result in better educational outcomes (e.g., Anders et al., 2012; Melhuish et al., 2008). Thus, we aimed to explore all relevant home-related factors	These two factors (Factor 5, Home Literacy Environment includes the number of books, bedtime reading routines, and unstructured play opportunities. Factor 6, Parental Literacy Attitudes includes enjoyment of reading and writing, confidence in language skills, and a negative association with screen time) did not contribute to our final regression models explaining children's STEM engagement scores with loose parts or control.<br>However, in order to show our rationale for including and exploring how children's home literacy environment and parents' literacy attitudes contributed to children's STEM outcomes, we included a paragraph on page 8, highlighting the need to account for the broader constellation of home experiences and learning opportunities

	comprehensively, as opposed to single out STEM-related ones. Also, instead of only asking questions regarding STEM-related activities, we included literacy.<br>When we conducted the exploratory factor analysis, we found six factors that meaningfully bunched up. Selecting the ones we thought may be relevant would have potentially biased our analysis; therefore, we included all factors.<br>References:<br><br>Anders, Y., Rossbach, H. G., Weinert, S., Ebert, S., Kuger, S., Lehrl, S., & Von Maurice, J. (2012). Home and preschool learning environments and their relations to the development of early numeracy skills. *Early childhood research quarterly*, *27*(2), 231-244.<br><br>Jang, W., Kwon, K. A., & Horm, D. (2024). The role of language and literacy skills in science learning from kindergarten to 5th grade: Mitigating gender, racial/ethnic, and socio-economic disparities. *Education Sciences*, *14*(9), 994.<br><br>Melhuish, E. C., Phan, M. B., Sylva, K., Sammons, P., Siraj-Blatchford, I., &	when examining play behaviours and cognitive development.

	Taggart, B. (2008). Effects of the home learning environment and preschool center experience upon literacy and numeracy development in early primary school. *Journal of Social Issues*, *64*(1), 95-114.	
10. Discussion – Discussion is clearly written but the results might change if the authors explore the two suggestions on the removal of Constructing Structures in the scale and the two factors in the factor analysis.	N/A.	We added a short section to the discussion on page 39 regarding how constructing behaviours can be nonverbal, and other STEM behaviours are mainly verbal. We also added a short section on page 8 in the introduction that explains and justifies why we included the two factors in our analysis.

Nature Communications Psychology
Manuscript COMMSPSYCHOL-25-0523

**Reviewer #1 Comments and Suggestions**	**Author Responses and Explanations**	**Revisions Implemented**
Thank you for providing an updated manuscript with clear responses to the reviewer comments. The authors have taken great care in revising the manuscript, making it a more coherent and well-rounded addition to the literature. I look forward to seeing it published.	Thank you for all the feedback provided.	No action was required.
**Reviewer #2 Comments and Suggestions**	**Author Responses and Explanations**	**Revisions Implemented**
I am generally satisfied with the revision, and the only comment I have would be to explain Figure 2 more in your Findings section.	Thank you for all the feedback and suggestions for improvement you provided.	We explained Figure 2 in more detail in the manuscript.
In addition, you would want to consider reducing the length of your findings as well as discussion to make it more concise.		We reduced the length of findings and discussion, and a professional editor helped us fine tune each section to make it concise.
